# Decision-Making with Predictions of Others’ Likely and Unlikely Choices in the Human Brain

**DOI:** 10.1523/JNEUROSCI.2236-23.2024

**Published:** 2024-08-23

**Authors:** Ning Ma, Norihiro Harasawa, Kenichi Ueno, Kang Cheng, Hiroyuki Nakahara

**Affiliations:** ^1^Laboratory for Integrated Theoretical Neuroscience, RIKEN Center for Brain Science, Wako 351-0198, Japan; ^2^Research Center for Life Sciences Computing, Zhejiang Laboratory, Hangzhou 311100, China; ^3^Support Unit for Functional Magnetic Resonance Imaging, RIKEN Center for Brain Science, Wako 351-0198, Japan; ^4^Laboratory for Cognitive Brain Mapping, RIKEN Center for Brain Science, Wako 351-0198, Japan

**Keywords:** computational modeling, dorsolateral prefrontal cortex, fMRI, posterior cingulate cortex, predicting other individuals, social decision-making

## Abstract

For better decisions in social interactions, humans often must understand the thinking of others and predict their actions. Since such predictions are uncertain, multiple predictions may be necessary for better decision-making. However, the neural processes and computations underlying such social decision-making remain unclear. We investigated this issue by developing a behavioral paradigm and performing functional magnetic resonance imaging and computational modeling. In our task, female and male participants were required to predict others’ choices in order to make their own value-based decisions, as the outcome depended on others’ choices. Results showed, to make choices, the participants mostly relied on a value difference (primary) generated from the case where others would make a likely choice, but sometimes they additionally used another value difference (secondary) from the opposite case where others make an unlikely choice. We found that the activations in the posterior cingulate cortex (PCC) correlated with the primary difference while the activations in the right dorsolateral prefrontal cortex (rdlPFC) correlated with the secondary difference. Analysis of neural coupling and temporal dynamics suggested a three-step processing network, beginning with the left amygdala signals for predictions of others’ choices. Modulated by these signals, the PCC and rdlPFC reflect the respective value differences for self-decisions. Finally, the medial prefrontal cortex integrated these decision signals for a final decision. Our findings elucidate the neural process of constructing value-based decisions by predicting others and illuminate their key variables with social modulations, providing insight into the differential functional roles of these brain regions in this process.

## Significance Statement

In daily life, to adjust our decisions, we constantly predict others’ choices, but the inherent uncertainty means we face multiple scenarios for different choices by others. Using computational modeling-based fMRI, we identified a network in three-stage computations for such decision-making. Amygdala signals represent predictions of others’ choices. These signals then interact with the posterior cingulate cortex and dorsolateral prefrontal cortex, representing the decision variables with the prediction of others’ likely and unlikely choices, respectively. Finally, these signals modulate the medial prefrontal cortex, influencing our final choices. These pivotal variables and their corresponding brain signals play a fundamental role in a broad range of social cognitive processes. Our findings shed light on underlying mechanisms for complex social interactions in human behavior.

## Introduction

In social circumstances, an outcome is dependent not only on our choices but also on those of other individuals. Furthermore, because our choices may have to be made before knowing others’ choices, the decision would involve a prediction of those choices. Previous studies broadly investigated this social capacity of the human brain from the social cognition and theory of mind perspectives (Schaafsma et al., 2015; Hackel and Amodio, 2018; H. Wu et al., 2020; Ho et al., 2022) and, more particularly, by using frameworks of value-based decision-making (Lee, 2008; Rangel et al., 2008). Some of the major questions are how various aspects of others are learned and encoded for prediction, such as their preferences, goals, beliefs, and abilities (Behrens et al., 2009; Suzuki et al., 2012; Wittmann et al., 2016; Munuera et al., 2018) and how different degrees of recursive thinking about others’ thoughts and actions are used (Hampton et al., 2008; Yoshida et al., 2008, 2010; Hula et al., 2018, 2021). In contrast, the present study aims to shed light on another aspect of the prediction. The prediction of others’ choices is by no means perfect, and our optimal choice may depend on the different choices of others. In such circumstances, our own decisions should take into account not only a single predicted choice of others but their multiple possible choices. Despite their importance in social cognition, however, the neural mechanisms and computations are not well understood. To address this issue, this study investigated neural correlates of the key decision variables by conducting a human functional magnetic resonance imaging experiment using frameworks of value-based decision-making, together with model-based fMRI analyses (Behrens et al., 2009; Dunne and O'Doherty, 2013).

We developed an experimental task in which participants chose between two options to gain a reward, with the potential rewards associated with each option varying according to the choices made by another person, which were not known to the participants at the time of their decisions. In some trials, a choice to be made by others was far more likely than another possible choice, whereas the likelihood of either choice was similar in other trials. We took advantage of these different kinds of trials to separately investigate the neural correlates for making one's own choices with more and less likely choices of others. Furthermore, in contrast to these main trials, we also conducted two types of control trials, one related to participants’ own simple value decision-making and another requiring the prediction of the choices of others alone. In particular, by contrasting the neural correlates of the main trials with those of the second control trials, we were able to distinguish the possible brain processes essential for the prediction of others’ choices to be used in making one's own choice from those merely involved in the prediction itself.

Previous studies indicated that a wide range of brain areas are involved in social cognition and decision-making (Ruff and Fehr, 2014; Yeshurun et al., 2021). Findings of our present study are broadly consistent with those of the previous studies, for instance, in that the amygdala is necessary for inferring others’ mental states (Hutcherson et al., 2015; Gangopadhyay et al., 2021). However, as shown later, our findings highlight the amygdala for using the predictions of other individuals’ choices to make one's own choices from among the brain areas related to these predictions per se. Further, from functional associations to the prediction signals (S. W. Chang et al., 2015; Alcala-Lopez et al., 2018), we show that the activations of both the frontal and parietal areas are related to each other for making one's own choices, constructed from predictions of others’ likely and unlikely choices.

## Materials and Methods

### Experimental design

#### Subjects

Our experiment involved 62 healthy, right-handed participants (27 women; age range, 20–28 years; mean age ± standard deviation, 21.3 ± 1.4 years) who provided written informed consent to take part in the study. We screened all participants to ensure that they had no prior history of neurological or psychiatric illness. The RIKEN Third Research Ethics Committee approved the study.

We report the results of 48 participants after applying the following exclusions. First, we excluded eight individuals based on their behavior in the two types of control trials. Our aim was to examine the behavior (and neural correlates) in the main trials for making one's own valued-based choices, which required prediction of the value-based choices of the other individual. Then, our behavioral inclusion criteria for the subsequent analysis using the main trials was the subject's demonstrations of their value-based choices in the respective control trials: the subject's behavior should at least be affected by both reward probability and magnitude, which were for the self in the control-choice trials to make their own choices and for others in the control-prediction trials to predict others’ choices. By implementing the inclusion criteria in the fit to behavior of our models (see below, Modeling for behavior in the control trials, for details), subjects were excluded when either *Δp* or *Δm* had the best fit to the behavior in either of the respective control trials. Second, to ensure appropriate BOLD signal acquisitions, we excluded 6 additional participants from the remaining 54 due to excessive head motion (>2 mm in any scan axis) during fMRI scans. Thus, the behavior and blood oxygen level-dependent (BOLD) signal analyses included data from 48 participants.

#### Experimental task

**Figure 1.**
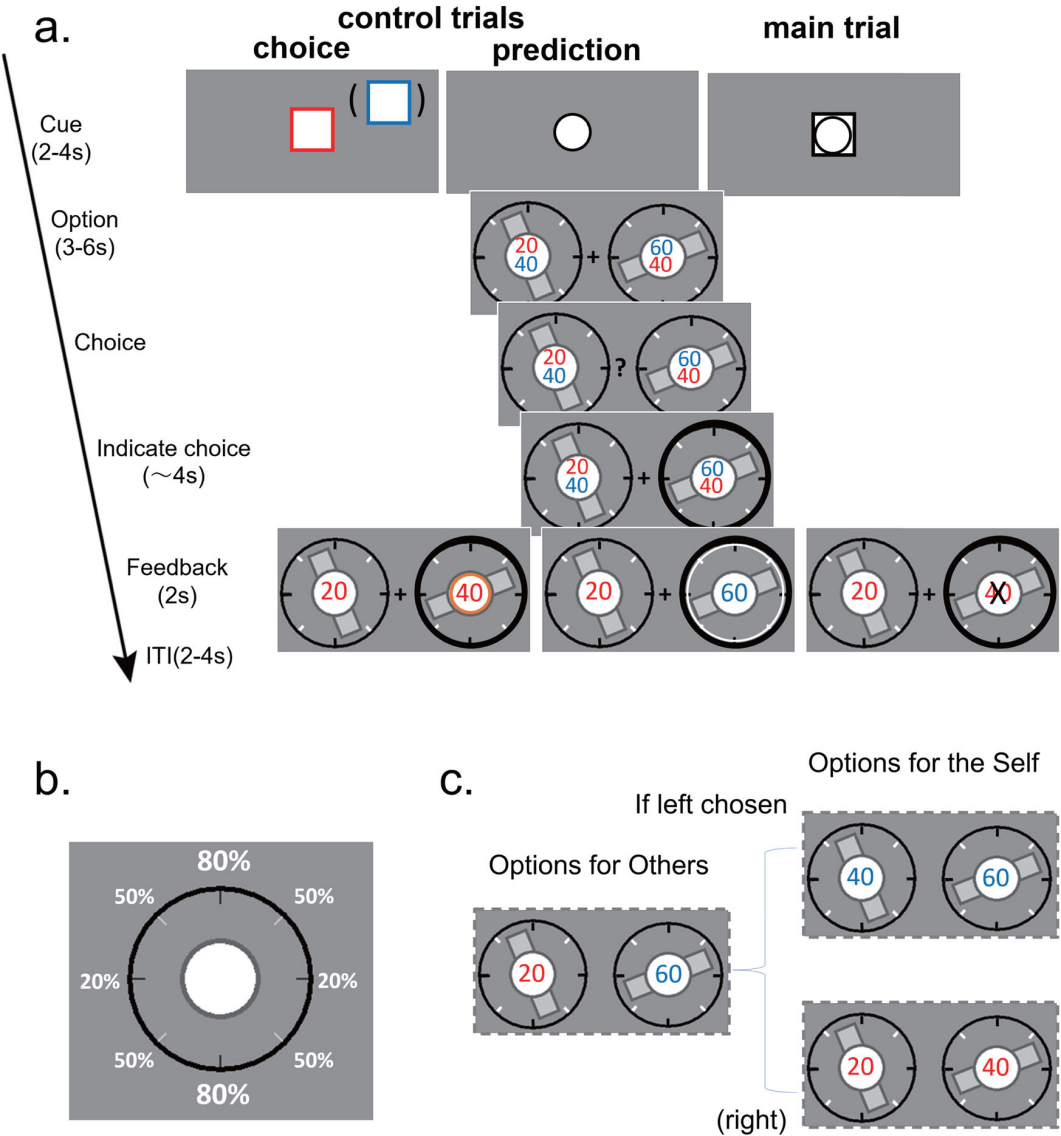
Experimental task. ***a***, The experimental task consisted of three types of trials, one main trial (cued by a rectangle with circle inside at the beginning) and two control trials (control-choice and control-prediction trials, cued by a rectangle and a circle, respectively). Subjects were instructed to choose between two options, each of which was associated probabilistically with rewards, in the main and control-choice trials to maximize their gains and in the control-prediction trials to predict another person's choice. Each option was provided with one orientation bar and two numbers in the center at the top and bottom. Reward probability was indicated by the orientation of the bar (see panel ***b***) and reward magnitudes were indicated by the number. In the main trial, which number at the top or bottom indicated the magnitude in a trial was determined by the other's choice (see panel ***c*** for details). In the control-choice trial, the number with the same color as the rectangular border of the cue would be rewarded. In this example, the cue is red, so the actual reward magnitudes were 20 and 40 for the left and right options, respectively. In the control-prediction trial, the reward magnitudes for others were always the top numbers regardless of the color. In this example, this is 20 for left and 60 for right. At the end of each trial, the outcome was shown. In this example, the subject chose the right option and was finally rewarded in the control-choice trial (indicated by an orange circle, 40 points) and in the control-prediction trial (correctly predicted the other's choice indicated as white circle on the right option and received a constant 30 points) but was not rewarded in the main trial (indicated by a black cross). All three trial types were interleaved in a block of trials. ***b***, Reward probability was sampled from five different probabilities, corresponding to different orientations of a bar. Here, in this orientation-probability map, a horizontal bar indicated the minimal probability of 20% while a vertical bar indicated the maximal probability of 80%. Bars with the three other possible orientations in the middle indicated probabilities of 35, 50, and 65%, respectively. The white numbers for reward probabilities here were not shown in the trials in the experiments, while the tick marks around the circle were always shown to help subjects to distinguish the different orientations. Different probability maps were set for self and others for a given participant, so that the same orientation bar can indicate different reward probabilities for the self and others. For details, see Materials and Methods, Setting of the bar orientation-reward probability map. ***c***, In the main trials, others’ reward probability was determined from the orientation-probability map for others, and others’ reward magnitudes were always shown by the numbers on top of the two options. Here, from the options in panel ***a***, the numbers were 20 for left and 60 for right. For self-options, for the option chosen by others, the reward magnitude used was the bottom number, whereas, for the option unchosen by others, the reward magnitude was the top number. Here, if the left option is chosen by others, the self-reward magnitudes are 40 for left and 60 for right. Otherwise, if the right option is chosen by others, the self-reward magnitudes are 20 for left and 40 for right. Therefore, to make better decisions for the self, subjects should predict others’ choices and figure out the reward magnitude for themselves.

Our experimental task comprised three types of trials: control-choice, control-prediction, and main trials (Fig. 1). All three types of trials were interleaved throughout the experiment. Each trial is a one-armed bandit task in which participants choose an option likely to result in a larger gain (number of points) by choosing such an option for themselves in the control-choice and main trials or choosing an option that they predicted others would choose in the control-prediction trials.

Each of the two options in the bandit task consisted of a reward probability indicated by the orientation of a bar and two reward magnitudes indicated by two numbers, top and bottom, in the center. We chose this configuration of options to minimize the number of visual elements to be presented and thus to reduce the overall processing burden when performing trials, especially for the main trial in which information on different reward probabilities and magnitudes of the self and others was required. A simple calculation example can explain the design. (1) Consider an option (in the experiment, we need two options but, here, we consider only one option for simplicity), and first assume that we like to separately indicate reward probability and magnitude differently for the self and other individuals; this leads to 2 × 2 = 4 elements simultaneously presented in one option. (2) Based on the aim, this study requires the choice of others to change the option of the subject in some aspect, and, in this task, we chose to let the others’ choice cause changes to a reward magnitude of the subject, which led to 4 + 1 = 5 elements. (3) We considered five elements in one option to be too many to ask (not to mention 10 elements in two options of a trial) and thus made a concerted effort to reduce the elements, leading to the current design. As detailed below, we chose to use only one visual element (not two) for the reward probability in one option, superimposing the probabilities for the self and other in one element. For reward magnitudes, we also superimposed magnitudes between the self and other, leading to two elements instead of three.

Reward probability, whether the reward magnitude of the option chosen by the participant in a trial was actually rewarded or not, was sampled from five different probabilities, represented by different orientations of the bar (Fig. 1*b*). This orientation-probability map was set as different for the self and others, so that the same orientation bar should indicate different reward probabilities for the self and others (see below, Setting of the bar orientation-reward probability map, for details). The two reward magnitudes (numbers) were always in two different colors, red and blue; the colors were used in the control-choice trials to indicate which reward magnitude should be paid attention to in each trial, and the randomization of the colors allowed us to examine decision variables in the trials, independently of the location (top or bottom) of the magnitudes in the configuration.

In the main trials of the experiment, the participants had to predict the decisions of others to make their own choices because the reward magnitudes for their own choices depended on the choices made by others. To predict the choices of others, the participants could use the reward probability of others, which was obtained from the orientation-probability map for others, and the reward magnitudes of others, which were always the numbers at the top of the two options. To make their own choices, the participants needed to use their own reward probabilities for each option, which originated from the orientation-probability map for the self. The reward magnitude in each option followed the choice of others: for the option chosen by others, the reward magnitude used was the number at the bottom of the two numbers in the option, whereas for the option unchosen by others, the reward magnitude was the top number. Therefore, to make better choices in the main trials and maximize their own rewards, the participants needed to predict the choices of others, thereby figuring out the reward magnitude for themselves, and then make their own choices.

For the control-choice trial, the color of the rectangular lines in the CUE phase indicated that reward magnitudes in the same color were to be used in the trial. Thus, in each trial, the participants had to make choices, using reward probabilities (according to the orientation-probability map for the self) and reward magnitudes in the color of the rectangular cue. The participants received a reward (points) of that magnitude if it turned out to be (probabilistically) rewarded for the option that they chose. For the control-prediction trials, the participants were told that others would make choices using the number at the top as the reward magnitude in each option, regardless of their colors. Thus, in each trial, the participants made choices while predicting others’ choices, considering the top numbers with the orientation-probability map for others. The participants received 30 points as a reward when their predicted choice matched the option chosen by others; otherwise, they received 0 points.

Each trial comprised five phases (Fig. 1*a*). The trial began with the CUE phase, during which a shape (a rectangle, a circle, or a rectangle inside a circle) was presented at the center of the screen for 2–4 s to indicate the type of trial that would follow. This was followed by the OPTION phase, in which a pair of options with a fixation point in between was displayed for 3–6 s. During the CHOICE phase, which began when the fixation point changed to a question mark, the participants made their choice by pressing a button with their right hand. Combined, the CHOICE and INDICATE phases lasted no longer than 4 s, which meant that the participants had to respond within this time window to be eligible for rewards. Failure to respond within this window resulted in no reward (zero points) for that trial. The INDICATE phase followed the CHOICE phase and involved the thickening of the black frame surrounding the chosen option to indicate the participants’ selection. The FEEDBACK phase began after participants made their choice and provided feedback on the outcome of their selection. In the control-choice and main trials, a circle or cross was displayed in the center of the chosen option for 2 s to indicate whether or not the chosen option was rewarded. In the control-prediction trial, the white frame around the option chosen by the other person was shown for 2 s, and the participants received a reward (with a fixed amount) if they made a correct prediction. The trial ended with a jittered intertrial interval (ITI) of 2–4 s before the next trial began. The timing jitters for the CUE, OPTION, and ITI phases were randomly generated from a uniform distribution. To maintain the participants’ motivation, the average points earned (total points divided by the number of trials) were displayed on the screen during the resting periods after each scanning session. Before the start of the experiment, participants were informed about the monetary compensation that they would receive, which was based on their average points earned during 60 randomly selected trials. Specifically, the monetary compensation per hour in yen was calculated using the formula 40 × (average points − 17) + 1,200, where 1,200 JPY was the base participation fee. This compensation scheme resulted in a mean total monetary gain of 9,000 ± 800 JPY (ranging from 7,000 to 11,000 JPY across all participants).

#### Experimental settings

##### Setting of the bar orientation-reward probability map

The setting of an orientation-reward probability map began with the choice of a starting orientation (i.e., angle). We used eight orientations (22.5° apart from each other). For convenience of explanation, we set the starting orientation as one of four possible orientations on the right of the circle (starting from the top, clockwise, 0°, 45°, 90°, and 135°); note that, for instance, an orientation bar of clockwise 180° is the same as a bar of 0° while a bar of 135° is the same as a bar of −45° (or +315°). Thus, it suffices to consider these four orientation bars to be the full circle. We set the starting orientation to correspond to the minimum reward probability. Then, the orientation orthogonal to the starting orientation (i.e., the angle of the starting orientation plus or minus 90°) should have the maximum reward probability. Between the minimum and maximum reward probabilities, we chose to sample three reward probabilities, equating a proportional change in angle to that in reward probability with an equidistance of three angles; that is, considering the starting orientation as 0°, we sampled three angles at 22.5°, 45°, and 67.5°. Taking these setups together, we decided to set the minimum and maximum reward probabilities as 0.2 and 0.8, respectively, and, accordingly, we also had three reward probabilities of 0.35, 0.5, and 0.65, corresponding to the three orientation bars of the three angles. In our experiments, we had to set two orientation-reward probability maps for the self and others. For this setting, we took the following strategy. We set the two maps as always having starting orientations with an offset of 45° (either clockwise or counterclockwise). Procedurally, given each participant, for the map for the self, we uniformly and randomly (with a constraint balancing across subjects) selected a starting orientation among four possible orientations and then set the map for others as having a starting orientation either +45° or −45° from the starting orientation for the self, counterbalanced across subjects. The reason for this strategy was to suppress the absolute value of possible correlations of choices given decision value (DV) differences for the self and others.

##### Generating trials

To make the best use of our experiments, we prepared experimental configurations of stimulus sets from an extensive sampling procedure via the following three steps. First, 10,000 candidate stimulus blocks, each of which comprised 44 trials, were generated as follows. Eight possible orientations encoded five reward probabilities both for self and for others, according to the orientation-probability maps (Fig. 1*b*, for example). Among the full 8 × 8 = 64 possible combinations of pairs of orientation bars between two options, we first excluded eight combinations of identical orientations. Then, the pairs with the same reward probabilities for self (six pairs) or for others (six pairs) were further excluded. Hence, the remaining 44 pairs of orientation bar combinations (i.e., reward probabilities) were used to constitute 44 trials in each candidate block. Regarding the reward magnitudes for each trial in the two options, three different whole numbers were randomly sampled uniformly between 1 and 99: two numbers for the top magnitudes and one for the bottom magnitudes (the bottom numbers were always the same for the two options). To be used in the control-choice trials, colors (one red, the other blue) were randomly assigned to the two numbers within each option and the color cue of each control-choice trial was also randomly selected.

Second, among the pool of 10,000 candidates prepared as described above, we selected the candidates by screening the distributions and correlations of potential decision variables. We generated the potential decision variables for each type of trial for each candidate block in the following way. As in the control-choice trials, we used the multiplication of reward probability and magnitude (determined by the cue color) for each option and considered their value difference as the potential decision variable. To examine their distribution and correlations, we used the absolute value difference (S_DV) and the value of the higher value option (S_CV; as if simulating a chosen value). As in the control-prediction trials, we similarly considered the value difference of others’ values and then used their absolute value difference and the value of the higher value option (O_DV and O_CV). As in the main trials, the prediction of others’ choices was treated in the same way in the control-prediction trials (i.e., O_DV and O_CV). Because there were two different pairs of options for the participants’ own choices, which were dependent upon the others’ choices, there were two possible sets of decision variables for the participants’ choices, and each set was modeled in the same way as in the control-choice trials; they were denoted by M_DV1, M_CV1, M_DV2, and M_CV2. Taking all of these variables of the three types of trial together, we had eight potential decision variables for each trial. We then screened 10,000 candidate blocks by evaluating their distributions and correlations within each candidate block. In terms of the distributions, two criteria were used: a block was excluded if the mean or standard deviation of any of the eight variables of the block fell out of the 90% confidence levels compared with their distributions across 10,000 candidate blocks or if any of the eight variables of the block did not follow a normal distribution (verified by Kolmogorov–Smirnov test). After these two exclusions, ∼3,000 candidate blocks survived. We further screened the blocks by examining the correlations in each block between several specific pairs of the eight variables (for DV, S_DV vs O_DV, O_DV vs M_DV1, O_DV vs M_DV2, and M_DV2 vs M2_DV2, and their corresponding CVs); blocks were excluded if any of their correlations were larger than 0.15; ultimately, 30 candidate blocks survived.

Finally, from the pool of these 30 candidate blocks, we generated a full set of stimuli for each participant. For each participant, eight blocks, where repeats were disallowed, were randomly selected from the pool and were then randomly assigned to roles of the three types of trials: two, two, and four blocks for the control-prediction, control-choice, and main trials, respectively. Half of the eight blocks (one, one, and two blocks for the three types) were used for the behavioral experiment and the other half for the fMRI experiment. This resulted in 44 control-choice trials, 44 control-prediction trials, and 88 main trials, giving 176 trials in total, for either the behavioral or fMRI experiment. The order of presentations was shuffled in the 176 trials so that the three types of trials were randomly interleaved in the experiment, which comprised four blocks of 44 trial presentations in the experiment.

##### Behavior of other individuals

Beforehand, we collected and prepared for the behaviors of others in the control-prediction and main trials in the following way. We recruited a group of participants who were independent from those who performed the main experiments. They performed a risk decision-making task, which would have been performed by the other person in the main experiment, using the same settings as the main experiment. All of these participants provided written informed consent and were paid based on their performance. Among these pooled participants, to simplify analyses of the main experiment, we selected the participants who made “rational decisions” relatively constantly, that is, those who usually chose the option with higher expected values: *m *× *p*, multiplication of reward probability by magnitude (confirmed by the same procedure of model selection for control-choice trials of the main experimental participants). This resulted in samples of 12 participants. For each participant, we randomly selected one individual among this pool (*N* = 12) as a “partner” for the given participant. The choice behavior of the selected partner was used in the training session and then throughout the behavior and fMRI sessions. Thus, when participants performed both behavior and fMRI sessions after sufficient training, they were already sufficiently capable of predicting the choices of the partner in the two sessions.

#### Experimental procedure

All of the main experimental participants participated in both the behavioral and fMRI experiments (outside and inside the scanner, respectively). Before the behavioral experiment, participants were familiarized with the experimental task. First, they were given instruction on the control-choice and control-prediction trials and subsequently practiced these trials, performing ∼5–10 trials for each trial type. Then, we held a learning session of trials for predicting others’ choices to ensure that the participants understood its nature and were able to predict others’ choices. The tasks in this learning session were similar to those in the control-prediction trial of the main experiment. Each participant had to predict the other's choice in each trial and received feedback, that is, correct or incorrect, but without earning any points. The session was continued until the prediction accuracy reached and was maintained at >85%. Participants could achieve this accuracy level in 50–100 trials. After the learning session, participants were instructed on the main trials and practiced ∼10–15 trials. In this practice session, to ensure that participants could understand the task of the main trial, an experimenter asked the participants about the rationale of their choice in two or three trials to see whether the participant could properly answer how to predict others’ choices and determine the information for the self. If the participant was confused, additional instruction (mostly the same instruction as before) was provided and more practice trials were added.

**Figure 2.**
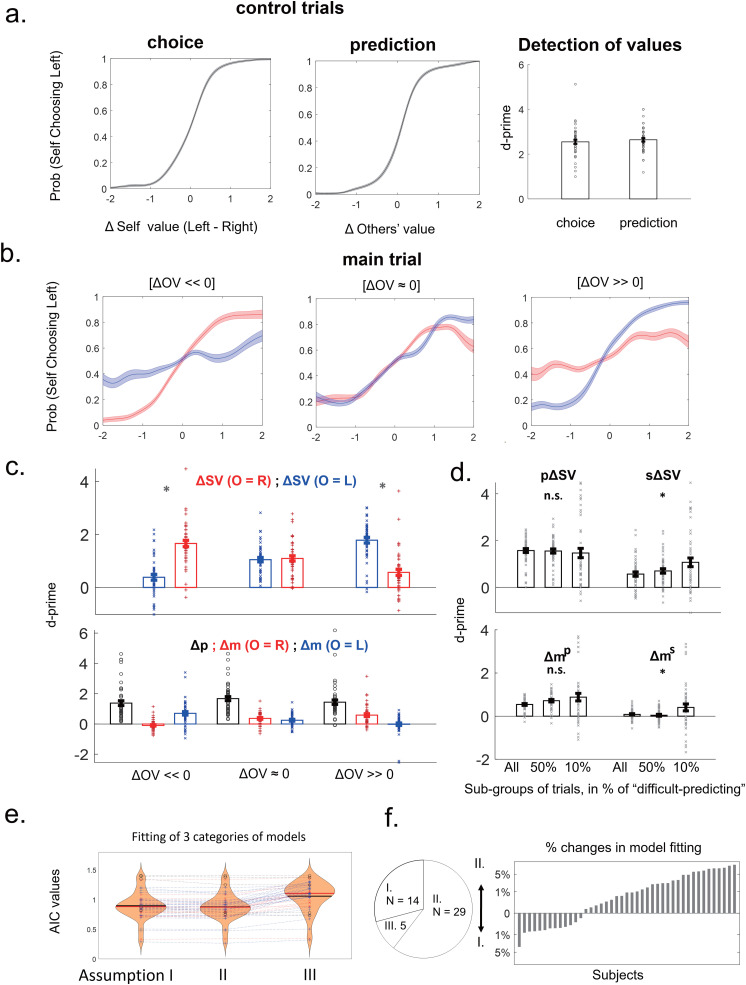
Behavioral results. ***a***, Control-choice and control-prediction trials. Subjects’ choices were modulated by the corresponding value differences. Left and middle panels, Their behaviors were plotted against the respective value difference, shown as the mean with standard error across subjects. Right panel, The effect size (*d’*) for detecting the higher values in the left options was presented according to the signal detection theory, wherein the bar and error bar indicate the means and standard errors, respectively, with dots indicating the *d’* of each subject (and similarly hereafter). The respective value difference showed significantly positive effects (*d’ *> 0, all *p* values <0.001) on their choice behavior in control-choice and control-prediction trials, with no significant difference in between (paired *t* test on *d’*; *t*_(47)_ = −0.749; *p* = 0.457). ***b***, Main trials. Subjects made their choices, relying on the use of value differences that were appropriate based on their predictions of others’ choices. Subjects’ choices followed two value differences for one's own rewards, as in each panel, plotted against *Δ*SV(*O* = *L*; blue) and *Δ*SV(*O*= *R*; red), articulated by the value difference for others, as shown across three panels, with three trial groups of almost equal sizes sorted by the magnitudes of *Δ*OV. The behavior was differentially well modulated by *Δ*SV(*O* = *L*) and *Δ*SV(*O* = *R*) when [*Δ*OV> 0] and [*Δ*OV < 0], respectively. All of the values were estimated by the best models from the control trials, and the differences were defined by the left minus the right. ***c***, Top, Statistics for panel ***b***; effect sizes (*d’*) of the six plots in panel ***b*** are shown; *, significant difference between the two effects ([*Δ*OV < 0], *t*_(47)_ = −6.954, *p* < 0.001; [*Δ*OV > 0], *t*_(47)_ = 8.525, *p* < 0.001). Bottom, The differential effects of the two value differences [*Δ*SV(*O* = *L*) and *Δ*SV(*O* = *R*)] were manifested through the difference in reward magnitudes. Effect sizes (*d’*) of the difference in reward probability (*Δp*) and difference in reward magnitudes [*Δm*(*O* = *L*) and *Δm*(*O* = *R*)]; *, significant difference between the two effects (for Δm, [ΔOV < 0], t(47) = −4.983, p < 0.001; [ΔOV > 0], t(47) = 6.067, p < 0.001). ***d***, Subject's choices were influenced by both types of the others’ value difference, especially when the prediction was difficult. Effect sizes (*d’*) of primary and secondary value differences (*pΔ*SV and *sΔ*SV, top panel) and primary and secondary reward magnitude differences (*Δm^p^* and *Δm^s^*, bottom panel) are shown for three subgroups of trials (all trials and the top 50% and top 10% of the most “difficult-to-predict” trials). *, significant with one-way repeated-measures ANOVA (for *sΔ*SV: *F*_(2,94)_ = 9.61, *p* < 0.001; for *Δm^s^*: *F*_(2,94)_ = 5.351, *p* = 0.006); n.s., no significant difference. ***e***, The fit to the choice behavior in the main trials is shown (AIC values, each of which indicates the averaged value across the trials for an individual participant) for the three categories of models based on Assumptions I, II, or III (abbreviated AI, AII, and AIII). AI, only primary values contributed to behavior; AII, both primary and secondary values contributed; AIII, without prediction of others’ choices. Violin plots indicate the distributions of all subjects (*N* = 48), with black and red lines representing the mean and median. To indicate each subject in the figure, a symbol was determined based on the best of the three models, namely, red “x”, blue “+”, or black “o” if the AI, AII, or AIII model was the best, respectively. The same symbol was used for each subject across the three models, connected by the line with the same color. Group-wisely, the AI or AII model was significantly better for fitting behavior than the AIII model (paired *t* test of AIC values: I vs III, *t*_(47)_ = −7.461, *p* < 0.001; II vs III, *t*_(47)_ = −8.688, *p* < 0.001) while the AII model was better for fitting behavior than the AI model (I vs II, *t*_(47)_ = 4.446; *p* < 0.001). These results were from the data of all experiments (fMRI + behavioral), and the same results were also found (*ps* < 0.001) by using only the data from the fMRI experiments. ***f***, Left panel, Numbers of the subject best fit by each model. Right panel, Percent changes of the difference in fit between the AI and AII models, sorted across subjects, not including subjects who were best fit by an AIII model. We observed a large variation but relatively gradual changes across the subjects, suggesting the behaviors were on a continuum rather than in distinct categories.

The main behavioral experiment was composed of four blocks and took ∼120 min, including the instruction and practice session described above. Each block consisted of 44 trials, resulting in 176 trials in a total of four blocks (88 for the main trials and 44 for each of the control-choice and control-prediction trials in total). All of these trials were randomly interleaved, as in the fMRI experiment. The timings of phases in the behavioral experiment were identical to those in the fMRI experiment, and the choice behavior of participants was recorded by the pressing of a button on the computer keyboard. After the behavioral experiment, participants were taken into the scanner room and performed the same task during fMRI scanning. The fMRI session took ∼100 min, including ∼60 min for scanning (see below, fMRI, for details). We reported the behavioral results in the main text by combining the trials of both the fMRI and behavioral experiments because we found no difference between all experiments (fMRI + behavioral experiments) and the fMRI experiments, as indicated by the model selection (see the legends of Fig. 2*e* and Table 1 for details).

**Table 1.** Results of model selection in the control trials

DV	*Δm*	*Δp*	*Δmp*	*Δm* + *Δp*
Control-choice				
Predicted accuracy	73.9 [69.9, 78.3]	70.5 [64.8, 74.4]	84.3 [80.7, 89.7]	89.8 [86.4, 92.0]
AIC	1.124 ± 0.162	1.157 ± 0.164	0.716 ± 0.237	0.588 ± 0.194
Comparison to the best model	*t*_(47)_ = 16.684^a^	*t*_(47)_ = 20.085^a^	*t*_(47)_ = 5.521^a^	Best model
Control-prediction				
Predicted accuracy	69.7 [66.9, 72.8]	79.3 [74.6, 81.8]	90.2 [87.4, 92.5]	90.2 [88.0, 92.6]
AIC	1.191 ± 0.119	1.010 ± 0.174	0.568 ± 0.213	0.582 ± 0.182
Comparison to the best model	*t*_(47)_ = 17.168^a^	*t*_(47)_ = 14.782^a^	Best model	*t*_(47)_ = 0.865^b^

All of these four decision variables had a significantly better fit to the choice behavior than chance in both types of control trials. “Predicted accuracy” is shown as the % of the correct prediction of the actual behavior in each model, with median [25 and 75% quantiles] across 48 subjects. For this, the predicted response, which was set as an option with a larger predicted choice probability by the model in each trial, was compared with the actual response in the trial. All of the eight cases were significantly greater than chance (*ps* < 0.001; Mann–Whitney *U* test, vs 50%). The *Δm* + *Δp* model was the best for control-choice trials and the *Δmp* model was the best for control-prediction trials. Please note that (1) AIC values are normalized (divided) by trial numbers and shown as means with standard deviations across all subjects (*N* = 48); (2) the free parameters (*β*) are omitted from the text but applied in the model fitting. For instance, *Δm* + *Δp* here represents the model of DV = *βp *× *Δp* + *βm *× *Δm* in the main text. Because we used real others who are relatively rational decision-makers, the difference between the self and others’ models, *Δm* + *Δp* for the control-choice trial and *Δmp* for the control-prediction trial, suggested that subjects can successfully learn to predict others according to others’ behavioral strategy. The above results were from the data of all experiments (fMRI + behavioral), and the same results were also found by using only the data from the fMRI experiments: the *Δm* + *Δp* model was the best for control-choice trials (AIC, compared with other models, *p* values <0.001) and the *Δmp* model was the best for control-prediction trials [n.s. comparing the *Δm* + *Δp* with the *Δmp* by AIC (*p* = 0.764) but *Δmp* was marginally significantly better than the *Δm* + *Δp* by BIC (*t*_(47)_ = 1.857; *p* = 0.069)].

a *p* < 0.001 for the *t *test with the best model.

b With this *t*-value, it is not statistically significant for a comparison between *Δm* + *Δp* and *Δmp* in terms of their AIC values (*p* = 0.391). However, based on their Bayesian information criterion (BIC) values, the *Δmp* model (BIC 0.624 ± 0.213) was significantly better than *Δm* + *Δp* model (BIC 0.667 ± 0.182), *t*_(47)_ = 2.526, *p* = 0.015. Thus, we chose the *Δmp* model as best model for control-prediction trials.

### Statistical analysis

#### Behavioral analysis and computational models

We constructed valuation and choice models based on value-based decision-making frameworks (Lee, 2008; Rangel et al., 2008; Behrens et al., 2009; Dayan and Nakahara, 2018). These models often underlie our behavioral analyses.

##### Behavior modeling

The participant's choice behavior was modeled in a value-based decision-making framework by using a SoftMax (sigmoid) function of the DV, given by the following:

$$q=\frac{1}{1+\exp\;(-\mathrm{DV})},$$

where *q* indicates the choice probability and DV indicates the value difference between the two options. In the behavior analysis, we used *q* as the probability that the option on the left was chosen and DV as the value of the left option minus that of the right option. In the fMRI analyses, we used *q* in the alignment of the choice actually made by the subject. For instance, in a trial when the right option was chosen by the participant, *q* and DV were the choice probability of the right option and the value of the right option minus that of the left option, respectively; in another trial, when the left option was chosen by the participant, *q* and DV were the choice probability of the left option and the value of the left option minus that of the right option in the trial, respectively.

The maximum likelihood approach was used to fit models to the behavioral data of the choice behavior. For each participant, the sum of the negative log-likelihood of choice probabilities for the options chosen by the participant (i.e., the probabilities *q* and 1 − *q* when the right and left options were chosen, respectively) was minimized using the “mnrfit” command in MATLAB (MATLAB R2017, MathWorks). The best-fitting model was the one that had a minimum of the sum of the negative log-likelihood, and the values of the free parameters were estimated from the best model and used in the subsequent analyses. To take into account the different numbers of free parameters for different models and compare their performance, we used distributions of Akaike's information criterion (AIC) values (Suzuki et al., 2012).

##### Modeling for behavior in the control trials

We examined the fit of four models to the choice behavior in the control-choice and control-prediction trials; DV was modeled by using *β_p _*× *Δp*, *β_m _*× *Δm*, *β_p _*× *Δp* + *β_m _*× *Δm*, and *β_mp _*× *Δmp*, respectively, where *Δ* indicates the left option minus the right option, *β* indicates weights for subscripts of each variable, and *m*, *p*, and *mp* indicate reward magnitude, reward probability, and the product of reward magnitude and probability, respectively. First, by model comparison with individual participants, eight participants were excluded from further analysis because their best model was *β_p _*× *Δp* or *β_m _*× *Δm* among the four models in either type of trials (see above, Subjects, for details). Then, by group-level comparisons, we found the best models (with statistical significances) to be *β_p _*× *Δp* + *β_m _*× *Δm* for the control-choice trials and *β_mp _*× *Δmp* for the control-prediction trials (Table 1). These models were then adopted as the basis for modeling parts of the prediction of others’ decisions and self-decisions, respectively, in the main trials.

We also plotted their choice behavior (Fig. 2*a*) using the decision variables estimated by the best models. The behavior was first plotted for each individual participant by applying smoothing by Gaussian filter (variance = 0.5) to the value difference (*x*-axis: left minus right) with the choice behavior (*y*-axis: left option, 1; right option, 0), and then the mean and standard error of the mean were calculated across all subjects.

We also examined *d’* (for choosing the left options) to determine the effect sizes of DVs on the choice behavior, based on signal detection theory. Specifically, *d’ = Z(Hit) − Z(False Alarm)*, where *Z(Hit)* is the *Z* score of the choice probability of *P*(Choosing left | left value > right value) and *Z(False Alarm)* is the *Z* score of *P*(Choosing left | left value < right value). Therefore, a larger positive *d’* indicates a greater effect of the corresponding DVs on the choice behavior, whereas *d’ *= 0 indicates random behaviors at the chance level. We computed *d’* for the (self) value difference and others’ value difference generated by their respective best models for the control-choice and control-prediction trials, respectively. One-sample *t* test against zero and paired *t* test between two control trials were performed.

Finally, we explain our modeling choices. In this study, we chose to use reward probability and magnitude in a crude (or linear) form. However, it might be questioned whether it would be better to use another, somewhat more polished, model such as the mean variance model to better reflect the intricate natures (e.g., risk) of value-based decisions. First, we preferred simplicity in terms of models, and we consider the current models sufficiently useful for capturing some essences of the behavior and neural correlates for the main interest of this study. Note that we do not claim that our models are the “best” models for capturing all of the intricacies of the behavior in our task. Second, simplicity was preferred for several reasons. The behavior studied in the main trials was already complex on its own, and it was thus better to avoid further unnecessary complexities. To assess the multiplicative and additive value formulations, the linear treatment of reward probability and magnitude gave us transparency. Particularly for analyzing one's own choices in the main trials, the additive form was used in which the linear treatment of the reward magnitude made the analysis particularly transparent, especially provided that the choice of others would change only the reward magnitude of the subjects. Given these factors, we preferred the current models with linear reward probability and magnitude.

Nevertheless, we examined the performances of other models for clarity and briefly mention the results (although the details are not shown here). The additional models examined were the mean variance model, two types of Prelec models, and the Tversky and Kahneman model and its extension, the Wu and Gonzalez model (Tversky and Kahneman, 1992; G. Wu and Gonzalez, 1996; Prelec, 1998). We replaced the multiplicative value as a whole by the mean variance model or replaced the probabilities in the multiplicative value by the variable produced by one of the other models. We compared their fit to the behavior in each of the two types of control trials among all of these models together with the four models described above (*β_p _*× *Δp*, *β_m _*× *Δm*, *β_p _*× *Δp* + *β_m _*× *Δm*, and *β_mp _*× *Δm*). The comparison was made in the same way for the comparison of the four models in the Results section (using AIC comparisons; in addition, we also conducted BIC comparisons). For the control-choice trials, the additive value model clearly stood out as the best model and was significantly better than all other models, even if multiple comparisons were taken into account. This was also true in BIC comparisons. For the control-prediction trials, there was no clear best model. In terms of AIC value, the model with the lowest value was the second-type Prelec model while the multiplicative value was lowest in terms of the BIC value. However, when these models were compared, their performance was not significantly better versus all of the other models. In AIC, three other models (mean variance, first-type Prelec, and Tversky and Kahneman) were close to the second-type Prelec model, followed by the multiplicative value. In BIC, the same three models (mean variance, first-type Prelec, and Tversky and Kahneman) were close to the multiplicative value, followed by the second-type Prelec and additive value models. Overall, we consider these results to support the use of the current models or, at the very least, to not suggest other models to be significantly better for the main interest of this study.

##### Analysis of behavior in the main trials

We analyzed the behavior in the main trials in two steps. First, using the best models for the control-choice and control-prediction trials (including the estimated parameter values of the models, obtained by fit to the control trials), we examined the behavior without directly fitting any models to the data in the main trials. Second, given the observations in the first step, we constructed different models, fit them to the behavior of individual subjects in the main trials, and then examined the behavior using the fitted models (with the parameter values of the models estimated by this fit).

In the first step, we calculated the others’ value difference (*Δ*OV, left minus right) and two types of the (self) value differences that were dependent upon others choosing the left or right option, denoted by *Δ*SV(*O* = *L*) and *Δ*SV(*O* = *R*). Note that, because we did not exactly know what the participant predicted about the choice of others in the main trials, these value differences were not based on the actual prediction of others by the participant but were based on a theoretical prediction of others’ choice by *Δ*OV. The theoretical prediction is to choose an option that has a larger expected value for others in that trial, which we call “theoretical choice” of others hereinafter. According to the theoretical prediction, others would choose the left option if the others’ value difference *Δ*OV > 0 or the right option if *Δ*OV < 0. To plot the choice behaviors (Fig. 2*b*), we first rank-ordered all of the trials according to the *Δ*OV and then split them into three subgroups with equal numbers of trials. For each of the three subgroups (*N* = 59, 58, and 59, respectively), we plotted the choice behavior (*y*-axis) separately with the two types of the self-value difference [*Δ*SV(*O* = *L*) and *Δ*SV(*O* = *R*), *x*-axis; Fig. 2*b*]. To probe the effect sizes of the self-values, *d’* was separately computed for each *Δ*SV in each subgroup of trials (Fig. 2*c*, top panel). For those six *d’* values, one-sample *t* tests against zero were first performed and, then, paired *t* tests between two *Δ*SVs were performed within each subgroup. Finally, the difference in *d’* for two *Δ*SV values in each subgroup were obtained using one-way repeated-measures ANOVA. Page trend tests were performed on these three differences. To investigate separable contributions to the choices by the reward magnitudes and probabilities, we conducted similar analysis using three pieces of decision variables for making one's own choices (Fig. 2*c*, bottom panel): difference in reward probabilities (*Δp*) and two differences in reward magnitudes in cases when others chose the left or right option [*Δm*(*O* = *L*) and *Δm*(*O* = *R*), respectively].

To further verify an effect of a difficulty in predicting others’ choices on their own choices, we analyzed the *d’* for their choices by using the contrast between others’ likely and unlikely choices (Fig. 2*d*). According to theoretical choices of others, we know that, if *Δ*OV > 0, others are more likely to choose the left option and, if *Δ*OV < 0, others are more likely to choose the right option. Using this information, for each trial, we classified the two self-value differences into those that were based on either others’ likely or unlikely choice, namely, as primary and secondary value differences, denoted by *pΔ*SV and *sΔ*SV, respectively. The primary value difference is the one more likely to be used, namely, *Δ*SV(*O* = *L*) when *Δ*OV > 0 and *Δ*SV(*O* = *R*) when *Δ*OV < 0, whereas the other difference is the secondary value difference, *Δ*SV(*O* = *R*) when *Δ*OV > 0 and *Δ*SV(*O* = *L*) when *Δ*OV < 0. To examine relationships of *pΔ*SV and *sΔ*SV with the participants’ own choices, we also grouped all of the trials according to the degree of difficulty, which was measured by the absolute others’ value difference, that is, |*Δ*OV|, with the smaller values indicating more difficult trials. We examined three trial groups: all of the trials and the top 50% and top 10% of the most difficult trials in predicting others’ choices (Fig. 2*d*, top panel). For those six *d’* values, one-sample *t* tests against zero were first performed and, then, paired *t* tests between two *Δ*SVs were performed within each subgroup. Finally, one-way repeated-measures ANOVA and the Page trend test were performed on each *Δ*SV across the three subgroups. We then repeated the same analysis with the difference in reward magnitudes (Fig. 2*d*, bottom panel), denoted as the primary and secondary reward magnitude difference (*Δm^p^* and *Δm^s^*).

##### Modeling for behavior in the main trials

The choice behavior in the main trials was fitted and thus examined by three types of models, each of which was derived based on a different assumption of how and whether their prediction of others’ choices was used for making one's own choices [Assumptions I, II, and III (AI, AII, and AIII); see below]. Separately for each participant, a model that best fit their behavior was chosen as the model for that participant, and the best collective models from all of the participants were used in the subsequent analyses.

In AI to AIII, assumptions on how the participant used their prediction of others’ choices played a critical role. First, both the AI and AII models assume that the predictions were used for the self-choice, but they differ in that the AI model assumed that only the primary prediction of others’ choice was used, whereas the AII model assumed that the secondary prediction of others’ choices was also possibly used, in addition to the primary prediction. By contrast, the AIII model assumed that no prediction was used. Second, we noted two characteristics of our models and experimental task. Given the results of the control trials, we adopted the form of additive value for one's own choice. Furthermore, in the main trials, others’ choices affected not the reward probability to the self but only the magnitude to the self. Taking the two characteristics together, the AI model, which is the valuation (or its difference) of the primary prediction for making one's own choice, is given in the additive form: probability difference + primary magnitude difference. This is crudely stated here and a more formal description is given below. Furthermore, if we consider the valuation with the secondary prediction for making one's own choice (which we introduce here only for an explanatory purpose because, in our tasks, there were only two cases: with primary prediction alone or with both primary and secondary predictions), it would be constructed in the same way, replacing primary with the secondary magnitude difference, where the probability difference was the same as the case with primary prediction. Therefore, for the AII model, when both predictions were used together, the additive value is given (crudely) by probability difference + primary magnitude difference + secondary magnitude difference. We formalize these differences below, while we note here that this understanding guided us to use the primary and secondary magnitude differences when analyzing subjects as a whole, as described in the previous section.

We developed our models using both reward probability and magnitudes, noting distinctions of others’ likely and less likely choices according to the theoretical prediction. Recall that for the option chosen by others, the reward magnitude used for the subject was the number at the bottom of the two numbers in the option, whereas, for the option unchosen by others, the reward magnitude was the top number. Therefore, for the theoretical choice (i.e., others’ likely choice), the participant should consider the reward magnitudes for oneself in the bottom number for the theoretical choice option and in the top number for the other option, respectively (Fig. 1*c*). We call the difference between these reward magnitudes, the primary reward magnitude difference (*Δm^p^*). The participant might consider the other sets of reward magnitudes, called the secondary reward magnitude difference (*Δm^s^*) hereafter: reward magnitudes in the top number for the theoretical choice option and in the bottom number for the other option. The secondary reward magnitude difference corresponds to the case for others’ less likely choice. This may happen, especially when the value difference of others was small, meaning that the participant's prediction did not happen to meet the theoretical choice (as suggested by Fig. 2*d*).

AI corresponds to the case in which the participant's prediction of the other's choice was the theoretical choice and their own choice was made according to the primary reward magnitudes (*Δm^p^*). These lead to a model for the prediction of others’ choices comprising DV*_o _*= *β_mp _*× *Δm_o_p_o_*, where the subscript *o* indicates that these variables are for others. In this model, the participant predicts the choice of others as the left option, if *Δm_o_p_o_* > 0; otherwise, the right option is predicted. Given this prediction, their own choice was modeled by the following:

$$\mathrm{DV}=\beta_{p}\times \Delta p+\beta_{m}\times \Delta m^{p},$$

where *Δp* is the difference in reward probability (for the self) and *Δm^p^* is the primary reward magnitude difference for the self.

AII corresponds to the case in which the participant takes into account not only the primary but also the secondary reward magnitude difference, when the prediction of the other's choice was relatively difficult. Thus, the participant's own choice was modeled by the following:

$$\mathrm{DV}=\beta_{p}\times \Delta p+\beta_{\mathrm{pm}}\times \Delta m^{p}+\delta_{x}\times \beta_{\mathrm{sm}}\times \Delta m^{s},$$

where *Δm^s^* is the secondary reward magnitude difference. The variable *δ_x_* is an indicator representing the difficulty of predicting the other's choice in the trial and was 1 or 0 for difficult and easy trials, respectively. Thus, the third term on the right side of the equation was included only for “difficult” trials. Because we could not definitively know which trial was difficult for a specific participant, we chose to use a grid search for fitting their behavior. First, we assessed the degree of difficulty in each trial by the absolute value of DV*_o_*, namely, |*Δm_o_p_o_|*, and sorted all of the main trials according to |*Δm_o_p_o_|* from small to large values, so that the more difficult trials were in the lower ranks. We then used a five-step grid search so that “*x*” percentages of the lower rank trials were considered difficult trials, and five cases of *x* were examined as 20, 40, 60, 80, and 100, denoted by *δ*_20_, *δ*_40_, *δ*_60_, *δ*_80_, and *δ*_100_; for example, *δ*_20_ became 1 if the trial fell into 20% of the lower ranked trials (i.e., the top 20% most difficult for the others prediction), and 0 otherwise. Thus, according to *δ_x_*, we had five models in AII, and each of them was separately fitted to the choice behavior of the subject.

AIII corresponds to the case in which the participant gave up inferring the choice of others and, instead, assumed that the choice of others was equal for the left and right options, so that DV is given by the following:

$$\mathrm{DV}=\beta_{p}\times \Delta p+\beta_{\mathrm{moL}}\times \Delta m\_oL+\beta_{\mathrm{moR}}\times \Delta m\_oR,$$

where *Δm_oL* and *Δm_oR* are the differences in reward magnitudes for the self when others chose the left and right options, respectively.

#### fMRI

##### Data acquisition and preprocessing

fMRI images were collected using a 4 T whole-body MRI system (Agilent Technologies) with a transverse electromagnetic volume coil as the transmitter (Takashima Seisakusho) and a 16-array head-shaped coil as the receiver (Nova Medical). For participants positioned in the scanner, visual input was provided via a mirror fixed on the coil, which reflects the images of a screen on the back side of the scanner, while a projector outside the shielded room projects to the screen through a waveguide on the wall. The participant used a button box to make their responses with the index and middle fingers of their right hands. BOLD signal was measured using a T2*-weighted echo planar imaging (EPI) sequence [668 volumes for each block; repetition time (TR), 1,076 ms; echo time (TE), 20.5 ms; flip angle (FA), 64°]. Twenty-five axial slices (thickness, 3.0 mm; gap, 1 mm; FOV, 192 × 192 mm; matrix, 64 × 64; resulting in voxel size, 3 × 3 × 3 mm) parallel to the anterior commissure–posterior commissure plane were acquired per volume. The EPI images were reconstructed by temporal generalized auto calibrating partially parallel acquisitions (TGRAPPA) algorithm (the acceleration factor, 2; Breuer et al., 2005). In addition, the alternating signal fluctuation by the TGRAPPA reconstruction was removed by our own software. The start of an experimental task was synchronized with the first EPI acquisition timing. Before, after, or between the functional runs, a set of high-resolution (1 mm iso) and a set of low-resolution (1.72 mm iso) whole-brain anatomical images were acquired using a T1-weighted 3D MPRAGE pulse sequence (TI, 500 ms; FA, 15°; with TR, 9.5 ms and TE, 3.7 ms for high-resolution scans and TR, 7.3 ms and TE, 2.5 ms for low-resolution scans). The low-resolution anatomical imaging slices were parallel to the functional imaging slices and were used to aid in the coregistering of the functional data to the high-resolution anatomical data. A pressure sensor was used to monitor and measure the respiration signal, and a pulse oximeter was used to monitor and measure the cardiac signal. The respiratory and cardiac signals were used to remove physiological fluctuations from functional images (Hu et al., 1995).

Functional and anatomical images were mainly analyzed using AFNI (Analysis of Functional Neuroimages; Cox, 1996) and MATLAB (versions 2017 and 2019; MathWorks). Functional images for each participant were preprocessed, which included slice time correction, removal of respiratory and cardiac signals, 3D motion correction, spatial smoothing with a Gaussian kernel (FWHM, 6 mm), high-pass temporal filtering (four cycles per run length), and temporal normalization (for each voxel, the signal of each epoch was divided by the temporally averaged signal). Then, images from all scanning sessions were connected. Anatomical images of each participant were transformed into standard Talairach space (Talairach and Tournoux, 1988). Functional images were analyzed in original space for each participant, and the resulting maps for statistical parameters were then normalized and resized according to the transformed structural images and thereby transformed into standard Talairach space.

##### Generalized linear model analysis

We applied a model-based analytical approach (O'Doherty et al., 2007) to analyze the BOLD signals by using generalized linear model (GLM) regression with two levels of analysis. In the first level, the BOLD signals of each participant were entered into the GLMs. In this approach, variables of interest to build the GLM were generated by using estimates from the behavioral models that were fit to the choice behavior of each subject. Hence, we created subject-specific design matrices containing the following regressors for the GLMs.

There were three GLMs: GLM1, GLM2, and GLM3. In GLM1, there were six or seven regressors for the variables of interest for each participant (please see above, Modeling for behavior in the main trials). There were two regressors, *Δp* and *Δm*, for the control-choice trials and one regressor, *Δmp*, for the control-prediction trials. For the main trials, regressors depended upon which model was selected by the behavioral fit, corresponding to AI to AIII described above: three regressors, *Δm_o_p_o_*, *Δp*, and *Δm^p^*, for AI; four regressors, *Δm_o_p_o_*, *Δp*, *Δm^p^*, and *δ_x _*× *Δm^s^*, for AII; and four regressors, *Δm_o_p_o_*, *Δp*, *Δm_oL*, and *Δm_oR*, for AIII. In GLM2, there were four regressors for the variables of interest: DVs for the control-choice and control-prediction trials, respectively, and DV*_o_* and DV for the main trials. In GLM3, all of the four regressors of interest in GLM2 were calculated by using SoftMax functions as choice probabilities, *q*. As described above, Behavior modeling, *Δ*, DV, and *q* were aligned to the behavioral choices in all of the GLMs (chosen vs unchosen options, rather than the left vs right options in the behavioral analyses). All of the regressors for the variables of interest were modeled within the DECISION phase, which was defined as the period from the onset of the OPTION phase until the participant responded in the CHOICE phase in each trial (Fig. 1*a*). After being mean corrected and normalized, all of the regressors were convolved with a canonical hemodynamic response function (HRF) for the entire duration of the DECISION phase, before being entered into GLM analysis.

We also examined BOLD response latencies different from the decision onsets. For this purpose, we constructed and used variants of GLM1, 2, and 3. Instead of convolution by the HRF signal in the original GLMs, each of the regressors for the variables of interest was convolved by five orthogonal HRFs with different delays; each HRF had a duration of 2 s; their onsets were delayed by 0, 2, 4, 6, and 8 s from the onset of OPTION; and they were orthogonalized in a sequential manner from shorter to longer delays. To probe the effect sizes of a variable of interest given different decision timings, the five corresponding convolved regressors were entered together into the same variant GLM. Therefore, three variant GLMs were created, generating five effect sizes for each variable of interest.

There were 21 regressors for variables of no interest in all of the GLMs. There were two regressors for each of the three trial types, which encoded the average BOLD responses for the CUE and DECISION phases, respectively, resulting in six regressors for the three types of trials. There were, across all of the trials, one regressor for the motor response of button pressing and two regressors for the outcome (one for the OUTCOME period and another for indicating if there was a reward or not, 1 or 0). These regressors of no interest were also convolved with a canonical HRF. Six motion correction parameters, indicating shift and rotation in each dimension in 3D space, were also entered as inputs to account for motion effects. Because they were entered as inputs with delays of 0 and 1 s for each motion parameter, there were 12 regressors for motion effects in total.

In the second level of the analysis, estimated effect sizes from the first-level GLM were entered into a whole-brain random-effects analysis for extracting significant brain activations for each regressor by using group-level statistics (i.e., group-wise one-sample *t* test). The brain regions with significant effect sizes for each regressor were reported based on corrected *p* values (*p *< 0.05), using familywise error (FWE) correction for multiple comparisons by permutation testing (5,000 permutations, using the AlphaSim command of AFNI, in which uncorrected *p* < 0.001 was mostly set, unless otherwise stated). To extract the contrast of effect sizes between two variables, random-effects analysis for comparing two effect sizes was performed by using group-level statistics (i.e., paired *t* test) within the activated clusters of one or two regressors, followed by a similar FWE correction procedure.

##### Temporal dynamics of neural signals

We also investigated the temporal features of the decision process in the main trial (Fig. 4*a–d*). Variants of the original GLMs were used to extract the time courses for each region of interest (ROI). Instead of using the HRF function, each variable of interest was separately convolved with nine base functions. Each base function had a single peak (value of 1) with values of 0 before the start time or after the end time. Spline interpolation (cubic smoothing splines) was used from the start time to the peak and from the peak to the end time. The peaks of the nine base functions occurred −3 to 21 s from the onset of the DECISION phase (with a 3 s lag between functions), and all started 3 s before the peak and ended 3 s after the peak. After convolution with the base functions, the nine regressors (for each variable) were entered into a single GLM. Therefore, nine parameters were estimated for each variable at each voxel. Time courses of brain signals for each variable of interest were then constructed by the weighted average of the nine effect sizes with the time course of the nine base functions. The time course of a ROI brain signal for each variable of interest was defined as the average time course of voxels that were within 5 cm in the 3D space of the voxels with the peak activations for the variable of interest. The time courses of the brain signals for each group of participants were obtained as the means and standard errors of the individuals’ time courses of the signals. The statistical significance for each time point was examined by using one-sample *t* tests against zero and independent samples *t* tests between groups.

##### Psychophysiological interaction analysis

Based on our GLM results, we used the psychophysiological interaction (PPI) analysis to probe functional connectivity, that is, the neural coupling of activated brain regions, by using the interaction term generated by signal fluctuations of one region given specific psychological conditions. PPI regressors were constructed as follows. First, ROIs were defined as the activated clusters in group-level activation maps from the GLM analysis. We then extracted the averaged BOLD time courses within a given ROI (as a physiological seed) for each participant based on their preprocessed BOLD signals. We then extracted the physiological signals from the seed by deconvolution of the time course of the seed with an HRF. For psychological seeds, we took a simpler approach (Wunderlich et al., 2012). Because we were primarily interested in how brain signals relating to decisions in one area were affected by other signals during a decision-making process, we simply set a variable encoding the time of the decision-making process, that is, the “DECISION” phase of trials, as the psychological seeds; we set the time as 1 for the DECISION phase and otherwise as 0, for all three types of trials. This seed was used for all of the PPI analyses except for the additional examination described in the Results; in that case, instead of the DECISION phase, we took others’ choice probability as the psychological seed. Using the physiological and psychological seeds, we also generated the interaction term. We first normalized each of the physiological and psychological terms to [0, 1] and then multiplied them by each other, further orthogonalizing the product to each of the two first-order terms. Next, the three terms (the interaction and the two first-order terms) were mean corrected and convolved with an HRF. To guard against possible confounding effects, we included not only the first-order terms of the interaction term as usual but also other regressors in the original GLM analysis.

We separately conducted ROI PPI and voxel-wise PPI analysis. For ROI PPI, we determined the target ROI based on the significant brain activations found by the GLM analysis and then conducted the PPI analysis. The effect size in the target ROI was tested using a one-sample *t* test against a null hypothesis of zero mean and paired *t* tests between different psychological seeds. For voxel-wise PPI, the activated regions were probed by using the interaction term for each voxel, in which the maps were analyzed as random effects by *t* test. Significant activations were determined and are reported as *p* < 0.05 (corrected), using small-volume correction (SVC) for which the ROI was used as mask, with FWE correction for multiple comparisons by the permutation test.

##### Individual differences in brain signals

We investigated differences in brain signals in the right dorsolateral prefrontal cortex (rdlPFC) with respect to individuals’ behavioral variations. In the original GLM analysis, the effect sizes for *Δm^s^* were available only for Group II (GII) participants, whose best models for the main trials followed AII and contained the regressor of *Δm^s^*. To extract the effect sizes of *Δm^s^* in the rdlPFC from Group I (GI) participants, additional GLMs were developed. The additional GLMs for the GI participants were defined by their best models only within AII models (note: the original best models for GI participants were based on AI). We thus extracted the rdlPFC activations for *Δm^s^* for each participant in GI and II (*N* = 43). We then examined the correlations of each participant's rdlPFC activations with differences in the AIC values of the behavioral fit of each participant between the AI and II models across all of the participants of GI and II and within each group. As a control, the same analysis was conducted with the posterior cingulate cortex (PCC) activation for *Δm^p^*. In GII participants, we first replicated two PPI results, the left amygdala or PCC activations with the rdlPFC activations, and then examined the correlations of the left amygdala activations for others’ decisions and the two neural couplings with the difference in AIC values.

### Code and data accessibility

The data and code used for analysis in this study are available on OSF (https://osf.io/ge69r/). Further information is available from the authors upon reasonable request.

## Results

### Behavior

To investigate how humans make their own choices that need to be guided by predicting the decisions of other individuals, we developed an experiment task involving three types of trials: main trials and two control trials called control-choice and control-prediction trials (Fig. 1; for details, see Materials and Methods). The main trials were our primary interest. In these trials, the available options for the subjects depended on the choices made by others, so that the subjects are needed to predict others’ choices to make their own choices. In contrast, in the control-choice trials, subjects made choices based on probabilistic reward outcomes without the need to predict others’ choices while, in the control-prediction trials, subjects indicated their choice of prediction about which option another person would likely choose. These two control trials were conducted to examine each of two processes, making one's own choice and predicting the choice of others, independently from each other, whereas, in the main trials, the two processes interacted, that is, the prediction of others’ choices informed what options to choose from, leading to one's own choices being made. Specifically, for the option chosen by others, the reward magnitude used for the subject was the number at the bottom of the two numbers in the option, whereas, for the option unchosen by others, the reward magnitude was the top number (Fig. 1*c*). Thus, the subject had to predict the choice of others to infer what reward magnitude the subject should use in their options and to then make their choices of the option.

#### Subjects making decisions when predicting others’ decisions

We first present the analyses of the choice behavior by the subjects in the two types of control trials, partly for ease of understanding and, more importantly, because we had planned to use the best model of each process to analyze the choice behavior in the main trials.

We found that their behavior was strongly influenced by differences in expected rewards (i.e., values) for the subject and others. To conduct these analyses, we adopted decision-making models based on value-based decision-making/reinforcement learning frameworks and conducted the models’ fit to the choice behavior: for making value-based decisions in the control-choice trials and for predicting others’ choices by emulating their value-based decisions in the control-prediction trials (see Table 1 for details). Our task involved the manipulation of reward probabilities and magnitudes separately. As a result, we examined four different decision variables to fit the models to the observed behavior. One variable was a usual value (magnitude × probability) while the other two variables were controls that used only either the magnitude or probability variable. The last variable involved the use of both variables in an additive way (i.e., not in a multiplicative way, as in the first variable; see Materials and Methods, Behavior modeling for details). This additive valuation was examined because some previous studies indicated that, when both reward probabilities and magnitudes were separately manipulated, such additive treatment might better fit the choice behavior than the “usual” value obtained by multiplication of the two variables (Rutledge et al., 2014; Blain and Rutledge, 2020). We refer to this additive variable as the “additive value” when it was necessary to distinguish it from the usual value variable (which could also be called the “multiplicative value”). We tested these four decision variables with reward probability and magnitude for the self in the control-choice trials and with reward probability and magnitude for others in the control-prediction trials.

Indeed, all of these four decision variables had a significantly better fit to the choice behavior than chance in both types of control trials (Table 1). In the control-choice trials, we found that choice behavior was best modulated by differences in additive values between two options for the subject (as shown in Fig. 2*a*, left panel, and confirmed as the best variable among the four different decision variables; Table 1). In contrast, for the control-prediction trials, choice behavior was best modulated by differences in multiplicative values between two options for others (Fig. 2*a*, middle panel; Table 1). With these best models, analysis based on signal detection theory (Fig. 2*a*, right panel) also confirmed that the effect sizes of the detection of higher values were significantly positive both in the control-choice trials (*t*_(47)_ = 27.033; *p* < 0.001) and in the control-prediction trials (*t*_(47)_ = 33.665; *p* < 0.001), with no significant difference between them (*t*_(47)_ = −0.749; *p* = 0.457). These results together suggest that the choice behavior of the subjects was well approximated by the use of value-based decisions for the self and of the prediction of others’ choices in the control-choice and control-prediction trials, respectively.

Notably, the best-fitting model differed between the two control trials: the additive value difference for the subjects’ own choices in the control-choice trials and the multiplicative value difference for the prediction of others’ choices in the control-prediction trials. We may wonder about the cause of the discrepancy in the best fitted model between the two cases and why the same additive value was not the best fit for predicting others’ choices. We did not pursue this issue further in this study, given the following observation. We had preselected a pool of other individuals whose behavioral choices were predicted by the subjects in the control-prediction trials. The selection was made to collect individuals whose choices were relatively aligned with the multiplicative value (see Materials and Methods, Behavior of other individuals for details). Given this fact, we consider it reasonable that the subjects’ behavior was best fit by the multiplicative rather than the additive value for predicting others’ choices. Additionally, although the discrepancy itself may be mostly due to our experimental setup, we also considered the discrepancy to support the subjects’ faithful prediction of others’ choices in the control-prediction trials, beyond their tendency for their own choices (additive value). Although not pursued in this study, a question remains whether the additive value difference would be a better model for predicting the choices of others in the face of a wide variety of other individuals when reward magnitudes and probabilities are independently manipulated.

We then proceeded with analyzing the choice behavior in the main trials, which involved both components (i.e., making one's own choices and predicting the choice of others). Given the results of the control trials described above, we adopted the additive value difference for one's own choices and the multiplicative value difference for the prediction of others’ choices. The analysis of the main trials in this section was based on these models (including parameter values estimated by the fit to the behavior in the respective control trials). In the next section, we use different models constructed from the two forms of valuations, fit these models (and thus their parameters) to the behavior of individual subjects in the main trials, and then examine a variety of behaviors.

We first aimed to demonstrate that subjects made their choices by relying on the use of value differences that were appropriate based on their predictions of others’ choices. To begin with the analysis, we noted two types of potential value differences for the subject in each trial, corresponding to when others chose the left or right option. We denoted these differences *Δ*SV(*O* = *L*) and *Δ*SV(*O* = *R*), respectively. We observed that the subject tended to use *Δ*SV(*O* = *L*) when others were more likely to choose the left option and *Δ*SV(*O* = *R*) when others were more likely to choose the right option. We categorized the main trials into three groups based on the difference in the value of others (denoted by *Δ*OV) between the two options—[*Δ*OV > 0], [*Δ*OV ≈ 0], and [*Δ*OV < 0]—with an almost equal number of trials in each group (*N* = 59, 58, and 59, respectively; see Materials and Methods, Analysis of behavior in the main trials for details). Figure 2*b* shows the choice behavior plotted against the two value differences. It was evident that the subject's value difference, either *Δ*SV(*O* = *L*) or *Δ*SV(*O* = *R*), better predicted the choice behavior when [*Δ*OV > 0] or [*Δ*OV < 0], respectively. However, when it was difficult to predict the choices of others ([*Δ*OV ≈ 0]; Fig. 2*b*, middle panel), both value differences were similarly predictive of the subject's choices. Signal detection theory analyses (shown in Fig. 2*c*, top panel) confirmed our findings. First, the *d’* values of all six cases (two types of value differences × three groups of trials) were significantly positive (all *p* values <0.008). Second, the effect size of *Δ*SV(*O* = *L*) was significantly larger than that of *Δ*SV(*O* = *R*; *t*_(47)_ = 8.525; *p* < 0.001) only in trials with [*Δ*OV > 0] while the effect size of *Δ*SV(*O* = *R*) was significantly larger than that of *Δ*SV(*O* = *L*; *t*_(47)_ = −6.954; *p* < 0.001) only in trials with [*Δ*OV < 0]. There was no significant difference between them when [*Δ*OV ≈ 0] (*t*_(47)_ = −0.480; *p* = 0.634). Additionally, there was a significant trend in the difference in the effect sizes between the two *Δ*SVs [*d’* of *Δ*SV(*O* = *L*) minus *d’* of *Δ*SV(*O* = *R*)] across the three groups of trials (one-way repeated-measures ANOVA; *F*_(2,94)_ = 54.867; *p* < 0.001; Page trend test; *L* = 642; *p* < 0.001).

Second, we examined the above findings on a finer scale using reward magnitude differences. For this, please note two characteristics of this study: (1) we used the additive form of valuations for one's choice, given the results of the control-choice trials; and (2) in the main trials, the choices of others impacted the subjects’ options only via the reward magnitudes of the subjects (Fig. 1*c*). Taken together, in the additive form, any differential effects of the two value differences [*Δ*SV(*O* = *L*) and *Δ*SV(*O* = *R*)] should manifest through the difference in reward magnitudes between the two value differences (see Materials and Methods, Modeling for behavior in the main trials). Therefore, we analyzed the corresponding distinct contributions of reward magnitudes and probabilities to the choice behaviors in the main trials. We conducted an analysis (Fig. 2*c*, bottom panel) that involved three decision variables for the subject's choices: the difference in reward probabilities between two options, denoted by *Δp*, and two differences in reward magnitudes, denoted by *Δm*(*O* = *L*) and *Δm*(*O* = *R*), respectively, for cases where others chose the left or right option. We discovered that the effect size (*d’*) of *Δp* was not significantly different between the two trial groups ([*Δ*OV < 0] and [*Δ*OV > 0]; *t*_(47)_ = 0.432; *p* = 0.667) or among the three trial groups (one-way repeated-measures ANOVA; *F*_(2,94)_ = 2.802; *p* = 0.066). However, for the reward magnitudes, we observed that the effect size of *Δm*(*O* = *L*) was significantly greater than that of *Δm*(*O* = *R*; *t*_(47)_ = 4.983; *p* < 0.001) only in the trials with [*Δ*OV > 0] and that the effect size of *Δm*(*O* = *R*) was significantly greater than that of *Δm*(*O* = *L*; *t*_(47)_ = 6.067; *p* < 0.001) only in the trials with [*Δ*OV < 0]. When [*Δ*OV ≈ 0], there was no significant difference between the two (*t*_(47)_ = 1.682; *p* = 0.100). This finding was further confirmed by a significant trend in the difference in effect sizes between the reward magnitude difference [*d’* of *Δm*(*O* = *L*) − *d’* of *Δm*(*O* = *R*)] across the three trial groups (one-way repeated-measures ANOVA; *F*_(2,94)_ = 35.08; *p* < 0.001; Page trend test; *L* = 626; *p* < 0.001). These results indicated that both reward probabilities and magnitudes played a role in decision-making by the subject but that the contributions of the reward magnitudes [*Δm*(*O* = *L*) and *Δm*(*O* = *R*)] were modulated by predictions of more likely others’ choices, corresponding to trials of [*Δ*OV > 0] and [*Δ*OV < 0], respectively.

Taken together, these results demonstrated that subjects were making decisions by predicting others’ choices, wherein an appropriate value difference of the subjects, at least one of the two types, was used. Next, we then asked the question, “Did the subject use only one type without considering the other type?” The subject's choice behavior may have been influenced not only by one type but both types, especially when the prediction was relatively difficult. To investigate this issue, we introduced a distinction between two value differences, called primary and secondary value differences, corresponding to the cases when the others make choices that are likely and unlikely chosen, respectively. We defined the primary value difference of the subject (denoted by *pΔ*SV) as that related to when others choose options likely to be chosen, that is, *Δ*SV(*O* = *L*) when *Δ*OV > 0 and *Δ*SV(*O* = *R*) when *Δ*OV < 0. In contrast, the secondary value difference (*sΔ*SV) is *Δ*SV(*O* = *R*) when *Δ*OV > 0 and *Δ*SV(*O* = *L*) when *Δ*OV < 0. The influence of the secondary value difference on choice behavior was examined in relation to the difficulty of predicting others’ choices. This difficulty was measured as the absolute difference in others’ values and also in the subsequent analysis. We used three groups of trials with respect to the difficulty: all trials, the top 50% most difficult trials, and the top 10% most difficult trials.

First, we found that when the subject used a prediction of others’ choices to make their own choices, they primarily relied on the primary value difference but also used the secondary value difference, particularly when the prediction was difficult. We analyzed the choice behavior with respect to *pΔ*SV and *sΔ*SV for the three groups of trials. We found that *sΔ*SV had a greater impact on choices in more difficult trials (Fig. 2*d*, top panel). Both value differences, *pΔ*SV and *sΔ*SV, had significant effects in all three trial groups (all six bars significantly different from zero, all *p* values <0.001 in Fig. 2*d*, top panel). However, the effect of *pΔ*SV did not differ across the three groups, indicating that its effect did not vary relative to the difficulty of predicting others’ choices (one-way repeated-measures ANOVA; *F*_(2,94)_ = 0.32; *p* = 0.728; Page test; *L*= 551; *p* = 0.995; paired *t* tests in three pairs; all *p* values >0.55). In contrast, the effect of *sΔ*SV increased significantly over the three groups of trials in the order of greater concentration on difficult trials (one-way repeated-measures ANOVA; *F*_(2,94)_ = 9.61; *p* < 0.001; Page test; *L* = 611; *p* < 0.001; paired *t* tests in three pairs, all *p* values <0.01). The effect sizes were larger for the primary value difference than for the secondary value difference in all trials and the top 50% groups (*pΔ*SV vs *sΔ*SV: *t*_(47)_ = 8.201, *p* < 0.001 and *t*_(47)_ = 7.102, *p* < 0.001, respectively). However, there was no significant difference between the two value differences in the top 10% group of trials (*t*_(47)_ = 1.576; *p* = 0.122).

Second, we demonstrated that the influence of the secondary value difference on choice behavior was mediated by the difference in reward magnitudes, particularly in difficult trials. We replicated the above analysis, instead by using the difference in reward magnitudes (Fig. 2*d*, bottom panel), which we refer to as the primary and secondary reward magnitude difference (denoted by *Δm^p^* and *Δm^s^*). We observed significant effects of *Δm^p^* (all *p* values <0.001) but found no difference in its effects across the three trial groups (one-way repeated-measures ANOVA; *F*_(2,94)_ = 2.843; *p* = 0.063). However, the effect of *Δm^s^* significantly increased with trial difficulty (one-way repeated-measures ANOVA; *F*_(2,94)_ = 5.351; *p* = 0.006). The effect of *Δm^s^* in the top 10% most difficult trials was significantly larger than zero (*t*_(47)_ = 2.547; *p* = 0.014) and was also significantly larger than the effects of all trials (*t*_(47)_ = 2.103; *p* = 0.041) and the top 50% most difficult trials (*t*_(47)_ = 2.594; *p* = 0.013). The effect sizes were larger for *Δm^p^* than *Δm^s^* in all three trial groups (*Δm^p^* vs *Δm^s^*: *t*_(47)_ = 8.448, *p* < 0.001; *t*_(47)_ = 9.130, *p* < 0.001; and *t*_(47)_ = 2.127, *p* = 0.039 for all, top 50%, and top 10%, respectively).

#### Modeling behavior in the main trials

Based on the above findings, we next investigated behavioral variabilities across individual subjects in the main trials. For this purpose, we fit to the behavior using different computational models. Our main interest was whether and how often the secondary value difference, in addition to the primary value difference, was related to the individual's choice behavior. We then used three computational models (see Materials and Methods, Modeling for behavior in the main trials for details). The first two assumption models (AI and AII) both assumed that subjects used predictions of others’ choices to inform their own decision-making. However, a critical difference between the AI and AII models is that the AI model used only the primary value difference while the AII model used both the primary and secondary value differences. Our secondary interest was to see whether or not most individual subjects would use predictions of others’ choices (which fall into either AI or AII models). Thus, our third model (AIII) assumed that the subject made no use of the predictions for making value-based choices. For fitting AII models to the behavior, we used a grid search strategy with five cases, where the secondary value difference was used only for 20, 40, 60, 80, or 100% of the main trials, based on the difficulty of the prediction of others’ choices (see Materials and Methods for details). We fitted all of these AI, AII (with the five cases, among which the best-fitting model was set as the AII model for the given subject), and AIII models to the choice behavior and compared their performance.

Analyzing the choice behavior by using these models, we found that most subjects incorporated predictions of others’ choices when making their own choices (i.e., best fitted by either the AI or AII models), with a large proportion of them using both primary and secondary value differences. First, when we compared the models’ fit to the behavior of all subjects, we found that the AI and AII models provided significantly better fits to the data than the AIII model (paired *t* tests of AIC values: I vs III, *t*_(47)_ = −7.461, *p* < 0.001; II vs III, *t*_(47)_ = −8.688, *p* < 0.001); a comparison of the AI and AII models revealed that the AII model was superior to the AI model (I vs II, *t*_(47)_ = 4.446, *p* < 0.001), as depicted in Figure 2*e*. These results suggest that the use of both the primary and secondary value difference was fairly common across the subjects. Second, we compared the models’ fit to each individual subject. Most subjects were better fitted by either the AI or AII models than the AIII model (*N* = 43 for either the AI or AII models vs *N* = 5 for the AIII model), indicating that most subjects (*N* = 43) examined in the analysis used predictions of the others’ choices to make their own choices. Of these 43 subjects, approximately one-third (*N* = 14, referred to as Group I, or GI) were best fitted by the AI model while the remainder (*N* = 29, Group II, i.e., GII subjects) were best fitted by the AII model (Fig. 2*f*, left). Thus, these results suggest that most of the subjects (GII or two-thirds) made their own choices using both the primary and secondary value difference, whereas a minority (GI) used only the primary value difference.

We next compared GI and II of the subjects in their choice behavior and their models’ fit to the behavior. First, we explored any possible further differences in choice behavior between GI and II subjects. However, we found no significant differences between the two groups: (1) in terms of the accuracy of the prediction of others’ choices in choice-prediction trials (median rates of correct prediction: GI = 0.881, range = 0.851–0.955; GII = 0.896, range = 0.736–0.966; I vs II, *Z* = −0.820; *p* = 0.412; Mann–Whitney *U* test), which was also confirmed by the lack of a significant difference in the effects (*d’*) of the detection of others’ choices (mean ± standard error: GI = 2.536 ± 0.419; GII = 2.698 ± 0.690; GI vs GII, *t*_(41)_ = −0.808; *p* = 0.424; independent samples *t* test); (2) in terms of the expected reward gain with their choices in main trials (median total expected points of the chosen option divided by the number of trials: GI = 26.62, range = 23.09–28.61; GII = 26.67, range = 23.08–30.43; I vs II, *Z* = −0.402; *p* = 0.688; Mann–Whitney *U* test), also confirmed by the lack of a significant difference in the expected reward difference per trial (expected reward points of the chosen options minus that of the unchosen option, divided by the total number, GI: median = 12.49, from 5.125 to 15.49; GII: median = 12.44, from 5.173 to 17.79; GI vs GII, *Z* = −0.350; *p* = 0.726; Mann–Whitney *U* test). These findings suggest that there were no differences in behavioral performance between the two groups. However, we caution that this could be due to our specific experimental task design. In more general settings, the use of secondary decision variables may lead to potential behavioral benefits. Nonetheless, the absence of differences in behavioral performance between the two groups allowed us to analyze the BOLD signals between GI and GII subjects, without being confounded by any differences in behavioral performance. Second, we examined variations in the relative fit between the AI and AII models among subjects of GI and II. For this, we analyzed the differences in AIC values between the two models, sorted by magnitude (Fig. 2*f*, right). We observed large variability in fit across subjects, suggesting significant variability in choice behavior. However, despite this variability, the difference in fit was relatively gradual across the subjects, without any sharp changes at any point and with increasing differences from left to right in the figure. This suggests that, although we identified two subject groups (GI and II) that differed in their use of secondary value differences, their behaviors were on a continuum rather than being two distinct categories.

### fMRI results

We conducted a computational model-based fMRI analysis to investigate the neural activities and processes underlying the prediction of others’ choices and one's own decision-making in the context of prediction. For analysis in the main trials, we used the best-fitting model among the AI–AIII models for each individual participant. We isolated each process to allow for a comparison of neural correlates between the main and control trials and also examined the interactions between fMRI signals for decision and prediction, as well as variations in activations that correlated with individual behavioral differences.

#### Brain activations by GLM analysis

##### mPFC and amygdala are respectively activated for self-decision-making and predictions of others’ decisions

**Figure 3.**
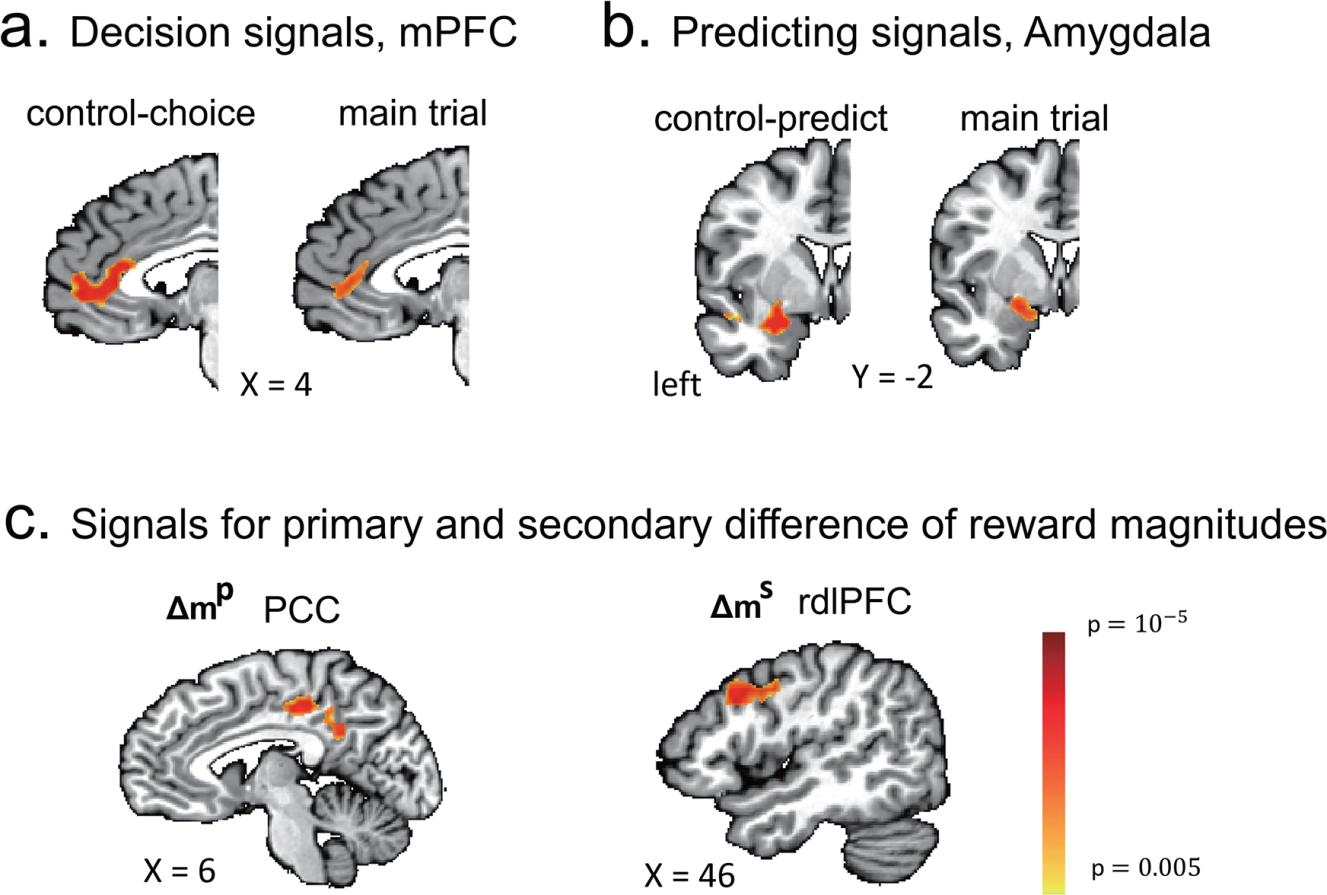
BOLD signals by GLM analysis. ***a***, Decision signals in the main and control-choice trials. Activations of decision values (*Δ*DV, the value of chosen options minus that of the unchosen ones, extracted by GLM2 for all Group I–III subjects) were found in the medial prefrontal cortex (mPFC). They survived at the FWE-corrected *p* < 0.05 level, and the same criteria are used in the following figures (or stated otherwise). For display purpose, here and hereafter, individual voxels in the activation map were first subjected to thresholding at *p* < 0.005 and then blurred at the resolution of anatomic imaging. ***b***, Prediction signals in the main and control-prediction trials. Activations of others’ choice probability for the chosen options (extracted by GLM3 using Group I and II subjects) were found in the left amygdala. Although the activation center in main trials is not visually within the left amygdala, the activation volume overlaps the left amygdala by eight voxels according to Talairach coordinates. ***c***, Signals for differences in reward magnitudes in the main trial. With GLM1 (using both Group I and II subjects for *Δm^p^* and using only Group II subjects for *Δm^s^*), the posterior cingulate cortex (PCC) was activated by *Δm^p^*, the primary reward magnitude difference (chosen vs unchosen) corresponding to the case in which others chose an option likely to be chosen. The right dorsolateral prefrontal cortex (rdlPFC) was activated by *Δm^s^*, the secondary reward magnitude difference corresponding to the other case in which others chose an option unlikely to be chosen.

First, we compared the neural correlates associated with decision-making in trials where participants made choices independently (i.e., the control-choice trials) versus those where they made choices based on others’ predictions (i.e., the main trials). We examined the activation of the subject's DVs, which is the difference between the value of the option chosen and that of the option not chosen. Our results revealed significant activations of BOLD signals in the medial prefrontal cortex (mPFC), as illustrated in Figure 3*a* (mPFC; control-choice trial, *x* = 1.8, *y* = 42.8, *z* = 6.3; main trial, *x* = 3.4, *y* = 42.4, *z* = 5.7, in Talairach space, left-posterior-inferior), as well as in other regions, such as the insula, middle temporal gyrus (MTG), superior temporal gyrus (STG), and inferior parietal lobule (IPL), in both the control-choice and main trials. However, no significant differences in activation were observed in these regions in a comparison of the control-choice and main trials (Table 2). These findings suggest that the neural activations associated with final decision-making for oneself are similar, regardless of whether one takes into account predictions of others’ choices.

**Table 2.** Areas exhibiting significant changes for self-decisions and predicting others’ choices in BOLD signals by GLM analyses

Activated clusters	Hemi	*x*	*y*	*z*	BA	*k*	Corrected *p*
Self-decision value							
Control-choice trial							
mPFC	LR	1.8	42.8	6.3	10/32	84	0.022
Insula	L	−44.9	1.1	1.3	13/22	145	0.006
Insula	R	50.8	−4.2	3.1	13/22	115	0.011
MTG, STG, IPL	L	−51.5	−44.7	58.5	22/39/40	336	<0.001
MTG, STG, IPL	R	55.8	−35.6	18.5	22/39/40	347	<0.001
Main trial							
mPFC	LR	3.4	42.4	5.7	10/32	36	0.002^a^
Insula/putamen	L	−28.7	−7.3	11.5	13	118	0.008
Insula	R	56	−4.5	10.2	13	54	0.035
MTG, STG	L	−50.1	−46.5	16.3	22/39/40	237	<0.001^b^
MTG, STG	R	54	−34.8	11.8	22/39/40	86	0.020^b^
Contrast of control-choice and main trials							
No cluster survived at corrected *p* < 0.05^c^							
Predicting others, others’ decision value							
Control-prediction trial							
mPFC	LR	0.4	49.4	5.5	10/32	137	0.007
Insula	L	−39	−0.3	9.4	13/22	209	0.005
Insula	R	43.5	−2.9	9.5	13/22	143	0.012
MFG, SMA	LR	1.1	−14.8	51.9	6	70	0.033
STG, IPL, TPJ	R	57.2	−30.1	20.1	40	189	0.006
Amygdala	L	−24.3	−6.4	−11.7	–	50	0.044
Main trial							
mPFC	LR	1.7	47.4	17.3	10/32	40	0.022^d^
Contrast of control-prediction and main trials							
No cluster survived at corrected *p* < 0.05^c^							
Predicting others, others’ choice probability							
Control-prediction trial							
Insula	L	−28.7	−5.9	9	13/22	44	0.049
STG, IPL, TPJ	R	57	−33.1	20.7	40	51	0.044
Amygdala	L	−30	−1.9	−13	–	119	0.015
Main trial							
Amygdala	L	−20.9	−2.7	−11	–	22	0.011^e^
Contrast of control-prediction and main trials							
No cluster survived at corrected *p* < 0.05^c^							

Activated clusters of variables were observed by voxel-wise GLM analysis of BOLD signals during the decision phase. *p* < 0.05, FWE corrected by permutation testing with whole-brain voxels (uncorrected *p* < 0.001, otherwise detailed in Notes a–e below). Activations by a whole-brain voxel-wise contrast were also analyzed. The stereotaxic coordinates are in Talairach space (LPI; see Materials and Methods, Generalized linear model analysis for details). BA, Brodmann area; k, number of voxels; Hemi, hemisphere. MFG, medial frontal gyrus; SMA, supplementary motor area; TPJ, temporoparietal junction.

a Corrected *p* = 0.002 (FWE correction within the activated ROI of the mPFC in control-choice trials, using an initially uncorrected *p* = 0.01, with signals at time 2–4 s, i.e., from the variant GLM) and they become a corrected p = 0.044 (FWE correction within the whole-brain activation map of the self-decision value in control-choice trials).

b Signals at time 6–8 s.

c No significant cluster survived with an uncorrected *p* = 0.005, by FWE correction, either in the whole brain or within activation maps for each type of trial or within each ROI.

d Corrected *p* = 0.022 (FWE correction within the activated ROI of the mPFC in control-prediction trials, uncorrected *p* = 0.01, signals at time 4–6 s) and they become a corrected p = 0.044 (FWE correction within the activation map of others’ decision value in control-prediction trials).

e Corrected *p* = 0.011 (FWE correction within the activated ROI of the amygdala in control-prediction trials, uncorrected *p* = 0.01) and they become a corrected p = 0.047 (FWE correction within the activation map of others’ choice probability in control-prediction trials).

Then, we investigated the neural correlates associated with the prediction of others’ choices, regardless of whether those predictions were used to inform one's own decisions. To do so, we used a regressor of others’ choice probability for the chosen option as the decision variable, rather than others’ DV, as the subject was only required to predict others’ choices in these trials, not their reward expectations or differences. Our results revealed that, in both the control-prediction and main trials, the choice probability had significant activations in the left amygdala (Fig. 3*b*; control-prediction trial; *x* = −30, *y* = −1.9, *z* = −13; main trial, *x* = −20.9, *y* = −2.7, *z* = −11). Furthermore, in the control-prediction trials only, significant activations were observed in the left insula, right temporoparietal junction (rTPJ), STG, and IPL. No significant difference was observed in any of these regions when we compared the control-prediction and main trials (Table 2). These findings suggest that the left amygdala activation stands out among the significant activations found in control-prediction trials because it was only significantly activated in the main trials. Additionally, when we examined the data using the DV of others instead of others’ choice probability, we found that the mPFC was activated in both the control-prediction and main trials, with no significant differences between the two (Table 2).

##### PCC and rdlPFC for primary and secondary value differences

We then explored possible neural processes unique to the main trials. In our behavioral analysis, we found that the subject's choice was influenced by both the primary and secondary value differences, with the effect most pronounced when we considered the primary and secondary differences in reward magnitudes (*Δm^p^* and *Δm^s^*; Fig. 2*d*). Furthermore, among all the subjects, the choice behavior of GI and GII subjects were influenced by the primary value difference, while that of GII subjects were also influenced by the secondary value difference. Accordingly, we investigated neural activations in response to *Δm^p^*, *Δm^s^*, and *Δp* during the main trials, using data from GI and II subjects (*N* = 43) for *Δm^p^* and *Δp* and only GII subjects (*N* = 29) for *Δm^s^*. First, we observed significant activations in the PCC in response to *Δm^p^* (Table 3, Fig. 3*c*), with two clusters surviving (one at *x* = −6.5, *y* = −27.1, *z* = 39.7, and the other overlapping with the precuneus at *x* = −0.6, *y* = −49.6, *z* = 31.5). In contrast, *Δm^s^* yielded significant activations in the rdlPFC (*x* = 46.4; *y* = 10.5; *z* = 34.8; Table 3, Fig. 3*c*). Analysis of the data from GI subjects as a control failed to reveal any significant activations in the rdlPFC in response to *Δm^s^*. Second, by contrasting activations in response to *Δm^p^* and *Δm^s^* with those identified by differences in reward magnitude in control-choice trials (*Δm^c^*), we identified clusters that survived in the PCC and rdlPFC (*Δm^p^* vs *Δm^c^* and *Δm^s^* vs *Δm^c^*, respectively; Table 3). Third, we found significant activations in response to *Δp* in the insula, MTG, STG, and IPL during both the control-choice and main trials, but no positive activations survived when we contrasted the main and control-choice trials (Table 4). Taken together, these findings suggest that the BOLD signals that correlated with the primary and secondary differences in reward magnitudes were specific to the main trials, rather than the control-choice trials and were activated in the PCC and rdlPFC, respectively.

**Table 3.** Areas exhibiting significant changes for self-reward magnitude differences in BOLD signals by GLM analyses

Activated clusters	LR	*x*	*y*	*z*	BA	*k*	Corrected *p*
Main trial							
Primary, *Δm^p^*							
PCC	L	−6.5	−27.1	39.7	24, 31	48	0.045
PCC, Precuneus	LR	−0.6	−49.6	31.5	7, 31	67	0.031
MTG, STG, IPL	L	−46.2	47	32.6	22/39/40	487	<0.001
STG, IPL	R	53.3	−49	29	22/39/40	138	0.007
Secondary, *Δm^s^*							
dlPFC	R	46.4	10.5	34.8	9	61	0.029
Control-choice trial, *Δm^c^*							
mPFC	LR	2.8	44.3	5.8	10/32	34	0.020
Insula	L	−50.9	−4.6	−6.7	13	16	0.046
Insula	R	54.8	−8.5	0.7	13	15	0.048
STG, IPL	L	−50.1	−48.9	18.3	40	74	0.003
STG, IPL	R	56.5	−40.5	19.9	40	79	0.003
Contrast of main trial > control-choice trial							
*Δm^p^* vs *Δm^c^*							
PCC	L	−5.7	−25.3	−40.7	31	19	0.035
PCC, precuneus	L	−9	−49.5	−30.1	31	12	0.008^a^
MTG, STG	L	−42	−52.8	−41.2	40	189	<0.001
STG	R	−52.1	−55.4	−30.9	40	54	0.003
*Δm^s^* vs *Δm^c^*							
dlPFC	R	46.2	10	34.8	9	61	<0.001

Activated clusters of variables were observed by voxel-wise GLM analysis of BOLD signals (FWE-corrected *p* < 0.05 by permutation testing). For main trials, an uncorrected *p* = 0.001 was first used and then corrected using whole-brain voxels; *Δmp*, signals at time 4–6 s of the decision phase; and *Δm^s^*, signals at time 6–8 s of the decision phase. For control-choice trials, uncorrected *p* = 0.005, corrected within the activation map of the decision value (for self-rewards) during the decision phase. Contrasted activations were obtained by using voxel-wise contrast between the main and control-choice trials; FWE-corrected *p* < 0.05, for which an uncorrected *p* = 0.005 was first used and then corrected within the union of activation maps (logical “OR”) between the main and control-choice trials. No cluster survived for the contrast of control-choice trial > main trial. PCC, posterior cingulate cortex; MTG, middle temporal gyrus; STG, superior temporal gyrus; IPL, inferior parietal lobule; dlPFC, dorsolateral prefrontal cortex; mPFC, medial prefrontal cortex.

a Corrected *p* = 0.008 using FWE correction within the activated ROI of the PCC in the main trial, while we had a corrected *p* = 0.063 using FWE correction within the union map between the control-choice and main trials.

**Table 4.** Areas exhibiting significant changes for self-reward probability and outcome in BOLD signals by GLM analyses

Activated clusters	Hemi	*x*	*y*	*z*	BA	*k*	Corrected *p*
Self-reward probability							
Control-choice trial							
mPFC	LR	1.3	43.2	5.9	10/32	64	0.004^a^
Insula	L	−45.6	2	4.3	13/22	106	0.014
Insula	R	49.7	−2.4	0.7	13/22	152	0.006
MTG, STG, IPL	L	−52.9	−45.3	18.6	22/39/40	217	0.002
MTG, STG, IPL	R	56.6	−34.2	21.8	22/39/40	213	0.002
Main trial^b^							
Insula/putamen	L	−49	0.1	5.1	13/22	67	0.004
Insula	R	46	−1.6	10.1	13	19	0.033
MTG, STG	L	−54.1	−43.8	12.3	22/40	40	0.016
MTG, IPL	R	52.9	−25.8	20.4	40	38	0.028
Contrast of control-choice trial > main trials							
Insula	R	−49.2	−0.5	−7.5	13	20	0.024
MTG, STG	L	−50.5	−49	18	40	65	<0.001
MTG, IPL	R	−58.3	−36.8	23.1	40	85	<0.001
Contrast of main trials > control-choice trial							
No cluster survived at corrected *p* < 0.05							
Outcome							
mPFC	LR	1.1	45.8	4.6	10/32	123	0.008
Nucleus accumbens	LR	−0.5	10.8	−2.9	–	67	0.03
STG	R	56.9	−28.2	13.4	41	56	0.039

Activated clusters of variables were observed by voxel-wise GLM analysis of BOLD signals during the decision phase. *p* < 0.05, FWE corrected by permutation testing with whole-brain voxels (cluster-defining threshold: *p* < 0.001, otherwise detailed in Notes a and b). Activations by a voxel-wise contrast for main trials versus control-choice trials were obtained [FWE-corrected *p* < 0.05, for which an uncorrected *p* = 0.005 was first used and then corrected within the union of activation maps (logical “OR”) between main and control-choice trials]. No cluster survived for the contrast of main trial > control-choice trial for self-reward probability. The stereotaxic coordinates are in Talairach space.

a Corrected *p* = 0.004, FWE corrected within activation maps for self-decision values in control-choice trials, uncorrected *p* = 0.005.

b The corrected *p* values reported in this table were based on the FWE correction within the corresponding ROIs of activations in control-choice trials (uncorrected *p* = 0.01). If corrected within the activation map for self-reward probability in control-choice trials, we have corrected *p* = 0.016, 0.100, 0.040, and 0.042 for each cluster.

#### Exploring the brain network of information processing in the main trials

The results of the GLM analyses suggested that there were neural activations specific to the main trials as well as common to both the main and control trials. In particular, the amygdala showed activity when participants were predicting others’ choices for making one's own decisions, while the mPFC was activated during the main trials for final decisions. The PCC and rdlPFC were found to be specific to the main trials and correlated with *Δm^p^* and *Δm^s^*, respectively. These findings suggest a possible three-stage process from predicting others’ choices to self-information (reward magnitudes) and then to actual choices. Although this does not rule out the potential for feedback or recurrent processing in these computations, it is possible that these activations are part of a cascaded three-stage process. To investigate this issue further, we examined the temporal aspects and functional connectivity of these activations.

##### Temporal dynamics of neural signals

**Figure 4.**
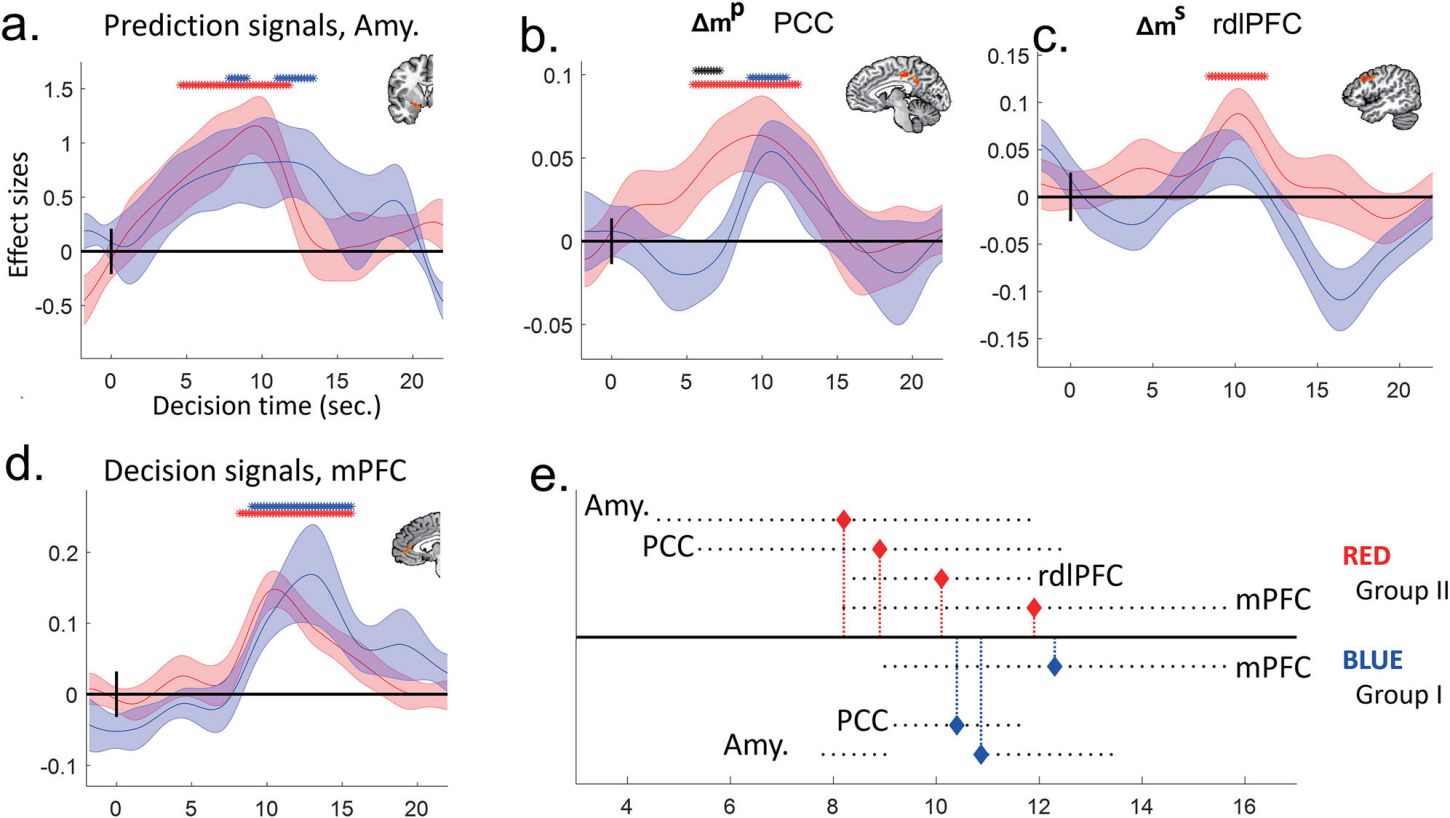
Temporal dynamics of brain signals. ***a–d***, Time course of brain signals in the main trials. For each of the two groups of subjects (Group I, blue; Group II, red), the mean and standard error were calculated and are shown as solid lines with shadings. Colored asterisks on the top parts of each panel indicate a significant positive difference against zero (one-sample *t* test; *p* < 0.05) for each group, while black asterisks indicate the differences between the two groups (independent samples *t* test; *p* < 0.05). The origin on the horizontal axis is set as the onset of the options. ***e***, Significantly positive time periods are summarized for each ROI of each group. The colored asterisks in panels ***a–d*** are shown here as small black dots. Colored diamonds indicate the average of all significantly positive times for each ROI.

To explore the temporal dynamics of the regions identified by significant activations in the above GLM analyses, we used temporally separated basis functions and combined them to understand their temporal behavior (see Materials and Methods, Temporal dynamics of neural signals for details). We extracted each individual's temporal activations using the best behavioral model, and then we collected group-level time courses and their statistics separately for GI and II subjects. Figure 4 illustrates the temporal dynamics for GI and II (represented by blue and red, respectively) for activations in the amygdala for predicting others’ choices, activations corresponding to *Δm^p^* and *Δm^s^* in the PCC and rdlPFC, and the mPFC activations for making one's own choices. The periods of significant positive activations are indicated by asterisks on the top of the figure. We examined the temporal order of the significant activation periods, in addition to their overall temporal curves (shown in Fig. 4), for probing the temporal order of the neural processes across different brain regions. Note that the analyses are inheritably limited due to BOLD signal temporal resolutions and should not be taken as definitive but as suggestive clues.

First, we observed that the temporal orders of the significant periods were *qualitatively* consistent with the three-stage–like computations suggested earlier (Fig. 4*e*). The average significant periods of the amygdala for predicting others’ choices were 10.87 s for GI (ranged from 7.8 to 9.0 and also from 11.0 to 13.4) and 8.2 s for GII (range = 4.6–11.8; Fig. 4*a*). When we also checked those in the mPFC for others’ DVs (data not shown), we found the average significant periods 11.0 s for GI (range = 7.2–14.8) and 6.1 s for GII (range = −0.2–12.4). For self-reward magnitudes, the average significant periods of the PCC for *Δm^p^* were 10.4 s for GI (range = 9.2–11.6) and 8.9 s for GII (range = 5.4–12.4; Fig. 4*b*), while those of the rdlPFC for *Δm^s^* were 10.1 s for GII (8.4–11.8) and there were no significant periods for GI (Fig. 4*c*). For final self-DVs, the average significant period of the mPFC, which occurred later than those periods, was 12.3 s in GI (range = 9–15.6) and 11.9 s in GII (range = 8.2–15.6; Fig. 4*d*).

We next compared the significant periods between the GI and II groups of subjects. The subjects in GII used both primary and secondary differences, whereas the subjects in GI only considered the primary difference. Hence, it is conceivable that the subjects in GII required more processing to reach their decision than those in GI, thus likely taking more time or starting to process earlier before the decision. Our comparison between GI and GII was consistent with this view (Fig. 4*e*): the timing of the neural processing tended to occur earlier in the GII than GI groups [GII vs GI: for predicting others, 8.2 vs 10.87 s (amygdala) and 6.1 vs 11.0 s (mPFC), for self-decisions, 8.9 vs 10.4 s (PCC) and 11.9 vs 12.3 (mPFC)], even though this observation should be understood only as suggestive due to the limited temporal resolution of BOLD signal dynamics.

##### Interactions of neural signals

We investigated interactions among the brain regions identified by our GLM analysis for the main trials through PPI analyses. Briefly, in our PPIs, we took a physiological seed from an activation of our interest. We defined a psychological seed simply as a time period of the DECISION phase (Wunderlich et al., 2012), unless otherwise stated. Thus, with these PPIs, we were able to examine whether the activation of our interest during a decision had significant functional connectivity with a target activation (i.e., a significant coefficient of the second-order term of the physiological and psychological seeds in the PPI analysis while regressing out the first-order terms and control variables; Materials and Methods for details). Specifically, we examined functional connectivity of amygdala activations with PCC activations and with rdlPFC activations; functional connectivity of PCC or rdlPFC activations with mPFC activations; and functional connectivities in each of both ways between PCC and rdlPFC activations. To ensure the robustness of our findings, we conducted similar analyses for the decision-making in the control trials. We applied two types of PPI analyses: ROI PPI, where the target ROIs were defined by the activations from our GLM results, and voxel-wise PPI (at the *p* = 0.05 level, FWE-corrected), which provided additional confirmation of the ROI PPI and visualized activated regions (see Materials and Methods for further details).

**Figure 5.**
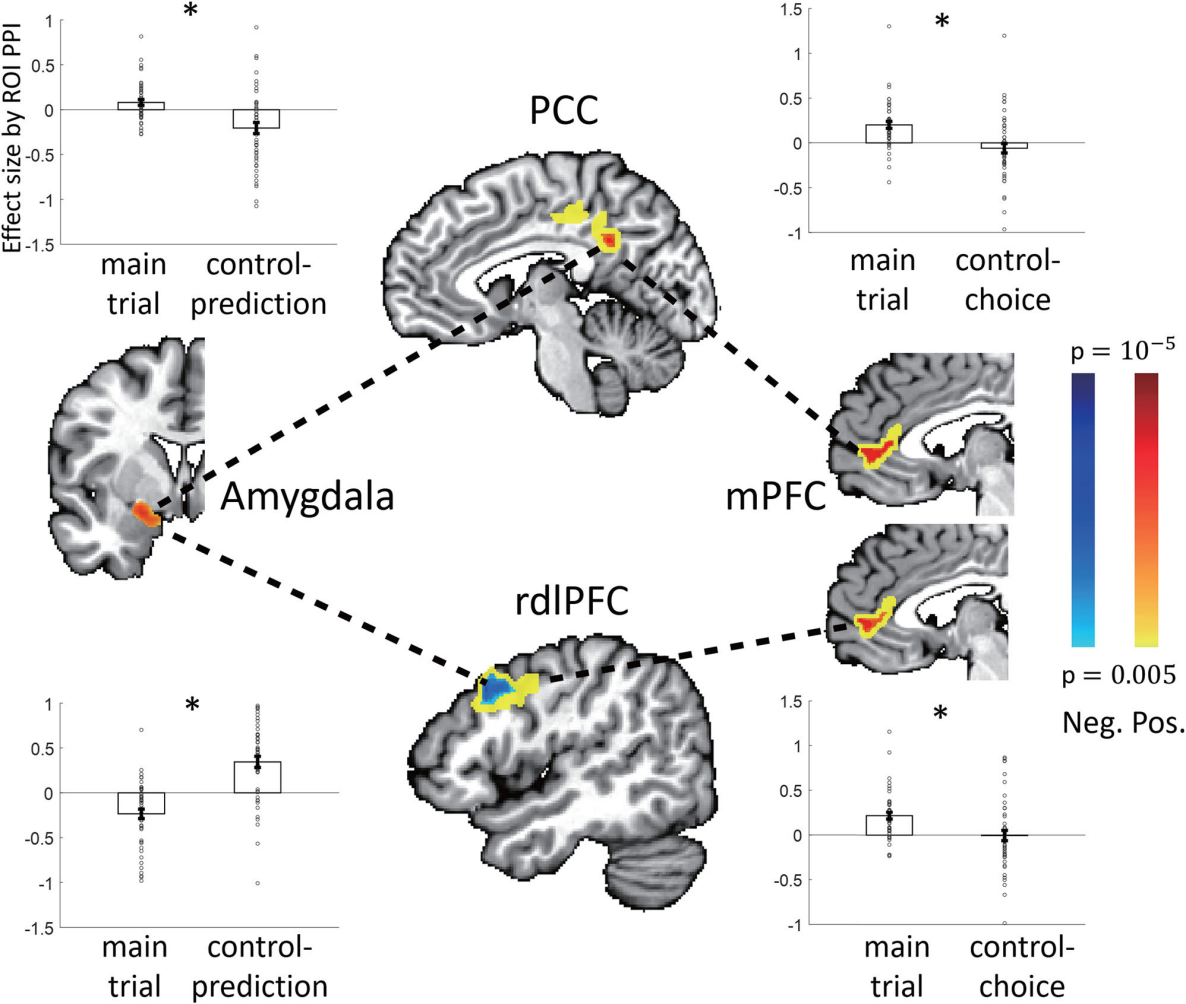
Functional connectivity by PPI analysis. Activation maps by voxel-wise PPI are shown here for the functional connectivities of the left amygdala with the PCC and the rdlPFC and of the PCC and rdlPFC with the mPFC, overlapped on ROIs generated from GLM activations (Fig. 3), which are shown here as yellow backgrounds. For display purposes, the activation identified by voxel-wise PPI is shown by an uncorrected *p* < 0.005, and the significance of the activation is tested using small-volume correction (SVC) within the target ROIs at the *p* < 0.05 level (FWE corrected). Bar plots show the ROI PPI results in the main and control trials, wherein * indicates significant differences between the main and control-prediction or control-choice trials. Top left and bottom left panels are the functional connectivities of the left amygdala with the PCC (*t*_(47)_ = 3.967; *p* < 0.001; main vs control-prediction trial) and with the rdlPFC (*t*_(47)_ = −6.968; *p* < 0.001; main vs control-choice trial), respectively. Top right and bottom right panels are the functional connectivity of the PCC with the mPFC (*t*_(47)_ = 3.955; *p* < 0.001; main vs control-choice trial) and of the rdlPFC with the mPFC (*t*_(47)_ = 2.699; *p* = 0.010; main vs control-choice trial), respectively.

Using left amygdala activations as the physiological seed and the DECISION phase as the psychological seed, we discovered significantly positive functional connectivity of the left amygdala activations with PCC activations during decision-making (ROI PPI, *t*_(47)_ = 2.551; *p* = 0.014; voxel-wise PPI, *x* = −4.5, *y* = −46.9, *z* = 30.8) but significantly negative functional connectivity with rdlPFC activations (ROI PPI, *t*_(47)_ = −4.508; *p* < 0.001; voxel-wise PPI, *x* = 46.3, *y* = 13.1, *z* = 35.7). As a control, we compared these PPI effects to the corresponding PPI effects in the control-prediction trials (using the left amygdala as the physiological seed and the DECISION phase in the control-prediction trials as the psychological seed; Fig. 3*b*, left). We found that the connectivity with the PCC in the main trials was significantly higher than that in the control-prediction trials (ROI PPI: main vs control-prediction, *t*_(47)_ = 3.967; *p* < 0.001; paired *t* test), whereas the connectivity with the rdlPFC was significantly lower than that in the control-prediction trials (*t*_(47)_ = −6.968; *p* < 0.001). These results are summarized on the left side of Figure 5 and in Table 5. Additionally, even when replacing the DECISION phase with others’ choice probability as the psychological seed, we discovered similar patterns of PPI effects: significantly positive connectivity with the PCC (*t*_(47)_ = 2.279; *p* = 0.027) and significantly negative connectivity with the rdlPFC (*t*_(47)_ = −3.376; *p* = 0.001).

**Table 5.** Areas exhibiting significant changes in BOLD signals by voxel-wise PPI analyses in the main trial

Seed	Target	*x*	*y*	*z*	BA	*k*	Corrected *p*
Left amygdala							
	PCC	−4.5	−46.9	30.8	31	24	<0.001
	Right dlPFC^a^	46.3	13.1	35.7	9	43	<0.001
PCC							
	Right dlPFC^a^	46.4	13	35.8	9	37	<0.001
	mPFC	4.5	42.5	4.5	10/32	28	<0.001
Right dlPFC							
	PCC	−4.9	−45.6	31.6	31	19	<0.001
	mPFC	3.8	43.5	4.6	10/32	32	<0.001

Activated clusters significant at the *p* < 0.05 level (FWE corrected) using voxel-wise PPI with the SVC at the cluster level by permutation testing within the corresponding target ROIs. The rest of the table format is the same as for Table 2. SVC, small-volume correction.

a The cluster of the right dlPFC showed significantly negative activation.

These results suggest that the neural activations for predicting others’ choices in the left amygdala are oppositely and significantly correlated with the activations for the primary and secondary reward magnitude differences in the PCC and rdlPFC, respectively. Although only correlational, this opposition is consistent with the view that the prediction signals in the left amygdala would help to balance the processing between the two areas. Neural signals in the left amygdala would be heightened when the prediction of others’ choices is more certain. Given our findings of their positive and negative connectivities on the PCC and rdlPFC, respectively, these heightened signals would contribute to an enhanced primary reward magnitude difference signal in the PCC and a suppressed secondary reward magnitude difference signal in the rdlPFC, resulting in greater reliance on the primary difference for decision-making. Conversely, when the prediction is less certain, lowered signals in the left amygdala would lead to less emphasis on the signals in the PCC as well as less suppression of the signals in the rdlPFC, resulting in more balanced use of both the primary and secondary differences.

Next, using PCC or rdlPFC activations as physiological seeds and the DECISION phase as the psychological seed, we found significantly positive functional connectivities of PCC or rdlPFC activations with mPFC activations during decision-making (with the PCC: ROI PPI, *t*_(47)_ = 5.186; *p* < 0.001; voxel-wise PPI, *x* = 4.5, *y* = 42.5, *z* = 4.5; and with the rdlPFC: ROI PPI, *t*_(47)_ = 5.584; *p* < 0.001; voxel-wise PPI, *x* = 3.8, *y* = 43.5, *z* = 4.6). To control for potential confounds, we contrasted these PPI effects with the corresponding PPI effects in the control-choice trials (using the same seed regions and DECISION phase in the control-choice trials as the psychological seed) and found that the connectivities of both PCC and rdlPFC in the main trials were significantly greater than those in the control-choice trials: the PCC (main vs control-choice trials, *t*_(47)_ = 3.955; *p* < 0.001) and the rdlPFC (*t*_(47)_ = 2.699; *p* = 0.010). These findings, summarized on the right side of Figure 5 and in Table 5, suggest that the activations of both the primary and secondary reward magnitude differences in the PCC and rdlPFC would have a positive association with the final decision signals in the mPFC.

Furthermore, we found significantly negative functional connectivity of the PCC activations with the rdlPFC activations provided the DECISION phase as the psychological seed (ROI PPI, *t*_(47)_ = −4.137; *p* < 0.001; voxel-wise PPI, *x* = 46.4, *y* = 13, *z* = 35.8; Table 5). This connectivity was also significantly lower than that in the control-choice trials (using the DECISION phase in the control-choice trials, ROI PPI, *t*_(47)_ = −4.12; *p* < 0.001). In contrast, we found significantly positive connectivity of the rdlPFC activations with the PCC activations (provided the DECISION phase as the psychological seed) in the main trials (ROI PPI, *t*_(47)_ = 2.673; *p* = 0.010; voxel-wise PPI, *x* = −4.9, *y* = −45.6, *z* = 31.6). However, this connectivity was not significantly different from that in the control-choice trials (*t*_(47)_ = 1.473; *p* = 0.147). These findings suggest that the PCC signals would be associated with suppressing signals in the rdlPFC in the main trials. This connectivity is conceptually consistent with earlier findings suggesting a suppressive effect of left amygdala activations on rdlPFC activations.

#### Neural processing in individual differences in behavior preferences

The activation of the rdlPFC was particularly noteworthy because it differed between GI and GII subjects who exhibited behavioral differences in their use of *Δm^s^* to make choices. Specifically, the rdlPFC was significantly activated by *Δm^s^* in GII subjects but not that in GI subjects, and the coefficient for *Δm^s^* in GLM was significantly larger in GII subjects than that in GI subjects (for GI subjects, using *Δm^s^* in their best-fitting AII model as a GLM regressor; see Materials and Methods; GII vs GI, *t*_(41)_ = 2.092; *p* = 0.043; independent samples *t* test). Therefore, we investigated the relationship between behavioral variations and neural activations. The behavioral indicator used was the difference in AIC values between AI and the best-fitting AII model (Fig. 2*f*), which reflects an inclination toward the use of *Δm^s^*.

**Figure 6.**
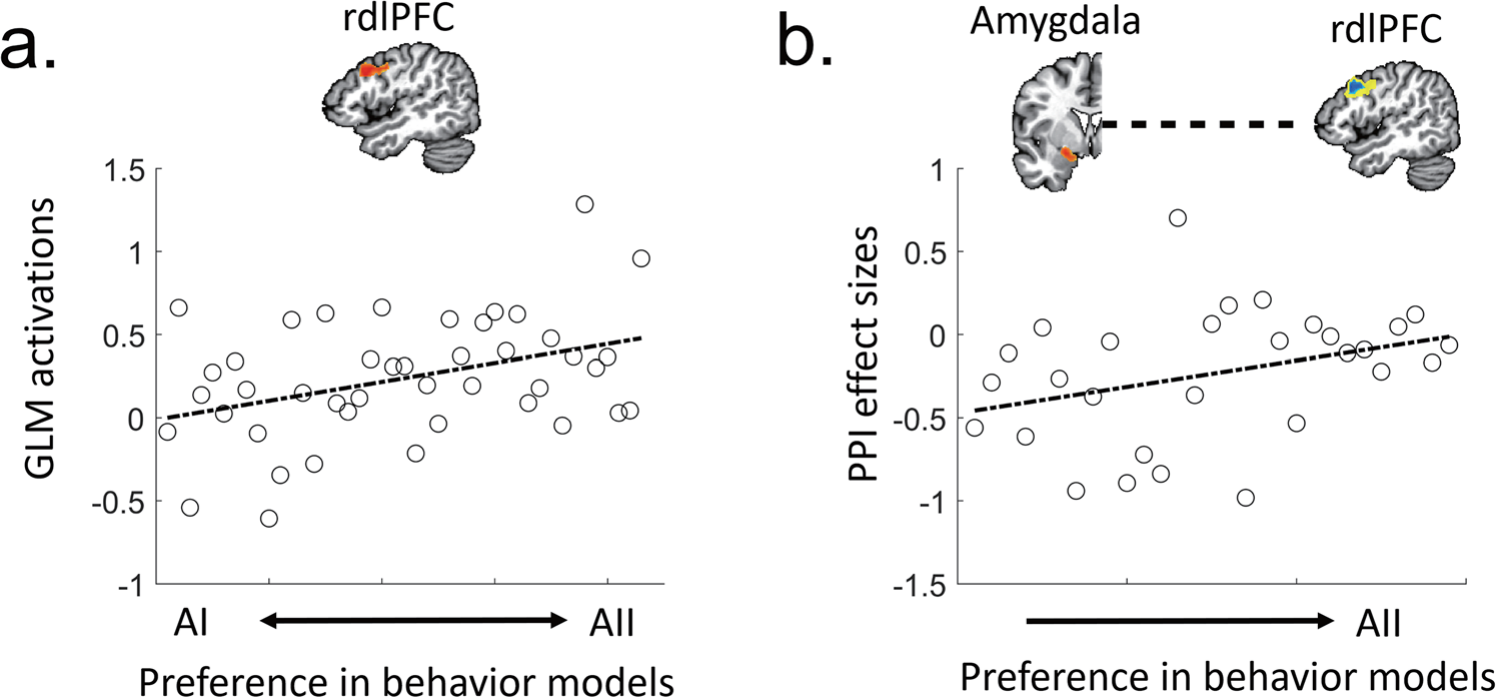
Brain signals across behavioral variations in individuals. ***a***, BOLD signals in the rdlPFC for the secondary reward magnitude difference (*Δm^s^*) are significantly positively correlated with individuals’ behavioral variations assessed by fitting variations between the AI and AII models (across all subjects from Group I and II; *ρ* = 0.337; *N* = 43; *p* = 0.028; by Spearman correlation). Behavioral variations were quantified by the AIC value difference between the AI and AII models and are rank-ordered in the figure. The rdlPFC signals were extracted from the 6 to 8 s duration from the onset of options, in line with our GLM findings. The same analysis of the signals of the 4–6 s duration was also significant (*ρ* = 0.397; *N* = 43; *p* = 0.009), thus confirming the current result. ***b***, Because the rdlPFC activations contributed to the use of the *Δm^s^*, we further investigated the relation between behavior and functional connectivities of the rdlPFC in Group II subjects. The PPI effect size of the left amygdala with the rdlPFC is significantly positively correlated with the individuals’ behavioral variations within Group II subjects (*ρ* = 0.400; *N* = 29; *p* = 0.033).

First, we found a significant positive correlation between neural activations in the rdlPFC and behavioral indicators for GI and GII subjects combined (Fig. 6*a*; *ρ* = 0.337; *N* = 43; *p* = 0.028; Spearman correlation). Second, when analyzed separately for each group, the correlation was marginally significant for GII (*ρ* = 0.346; *N* = 29; *p* = 0.066) and not significant for GI (*ρ* = −0.042; *N* = 14; *p* = 0.892). As a control, we conducted the same analysis with PCC activation for *Δm^p^* but found no significant correlation with the behavioral indicator for GI and GII subjects combined (*ρ* = 0.146; *N* = 43; *p* = 0.349), within GII (*ρ* = −0.075; *N*= 29; *p* = 0.699), or within GI (*ρ* = −0.266; *N* = 14; *p* = 0.357), nor a significant difference in activations between the two groups (GII vs GI, *t*_(41)_ = 0.686; *p* = 0.497). These findings suggest an overall tendency across all GI and GII subjects that rdlPFC activations correlate with the behavioral inclination toward the use of the secondary value difference. The data points in Figure 6*a* indicate very gradual changes across the *x*-axis rather than a sharp change. This is consistent with our earlier speculation of a behavioral continuum between GI and GII rather than the behavior between two groups being two completely separate categories.

Next, using only GII subjects, we examined the correlation of the behavioral indicator with two PPI connectivity effects on rdlPFC. First, using only GII subjects, we replicated two PPIs on rdlPFC activations in the earlier analyses with all subjects. The results were consistent with the earlier findings. There was significantly negative functional connectivity of the left amygdala activation on the rdlPFC activation and significantly negative functional connectivity of the PCC on the rdlPFC activation in the main trials (ROI PPI: left amygdala, *t*_(28)_ = −3.199, *p* = 0.003; PCC, *t*_(28)_ = −2.824, *p* = 0.009). Then, using the same behavioral indicator, we observed significant positive correlations between the behavior preference and both PPI connectivity effects (left amygdala: *ρ* = 0.400, *N* = 29, *p* = 0.033, shown in Figure 6*b*; also, PCC: *ρ* = 0.433, *N* = 29, *p* = 0.020). As depicted in Figure 6*b*, subjects who tended to use *Δm^s^* in their behavior, on the right side of the figure, had a PPI effect size by the amygdala near zero, whereas subjects on the left side had a negative PPI effect. This suggests that the suppression of the left amygdala on the rdlPFC activations was stronger for subjects who did not use *Δm^s^* in their behavior but was near zero for subjects who did use *Δm^s^*. Furthermore, we also found a significant negative correlation between the left amygdala activations for others’ choice probability and the behavioral indicator (*ρ* = −0.417; *N* = 29; *p* = 0.025). This finding is consistent with the previous results and further suggests that the potentially suppressing signals of the left amygdala on the rdlPFC tend to be weaker for subjects who tended to use *Δm^s^* in their behavior.

## Discussion

Using human fMRI experiments, we investigated behavior and neural correlates of decision variables in individuals when they predicted others’ choices to make their own choice. We demonstrated that they consider not a single choice but multiple predicted choices. Specifically, when others’ choices were easy to predict, information from the primary valuation—the primary reward magnitude difference—was utilized. When it was harder, however, additional information was used from the secondary valuation, the secondary reward magnitude difference. The extent of using only the primary or both information types varied across individuals and was affected by prediction difficulty. With these behavioral findings and computational model-based analysis of BOLD signals, we identified three prominent neural correlates: the left amygdala was correlated with others’ choice probability; the PCC and rdlPFC were related to the signals for self-decisions and modulated by the predictions of others’ choices (i.e., primary and secondary reward magnitude differences, respectively); and the mPFC was activated for subjects’ own choices.

The left amygdala signals were significantly correlated with the predicted choice probability of others. Our findings suggest that the amygdala (left) could be a unique area for using prediction to adjust one's own choices. Several brain areas (amygdala, TPJ, STG, and IPL) were significantly activated by others’ choice probability when simply predicting others’ choices in the control-prediction trials. However, in the main trials, which required use of the predictions for adjusting one's own choices, only the left amygdala was significantly activated. These left amygdala activations are broadly concordant with previous social behavior findings: the amygdala was associated with expectations about others and social influence generally, such as social attention, emotion recognition, interpersonal distance, social rank of faces, and social networks (Adolphs et al., 2005; Frith and Frith, 2006; Kennedy et al., 2009; Haruno and Frith, 2010; Baron et al., 2011; Bickart et al., 2012; Dal Monte et al., 2015, 2020; Kumaran et al., 2016; Wang et al., 2017). Similar signals have been observed in single neurons in nonhuman primates (Grabenhorst et al., 2019; Grabenhorst and Schultz, 2021).

Here we discuss the scope and limitations of this study using computational model-based analysis. The strength of the analysis lies in its examination of brain activations with key variables of the models or the assumed internal processes. Variable values varied across trials with different given information, and the changes were parametrically related across the variables in the process. Activations were found that correlated with such values of the variables. A parsimonious interpretation would be that an activation significant for each variable reflects the variable's values while such activations together correspond to the assumed processes. However, these interpretations require sufficient caution. Our univariate analysis found strong activations as chunks but did not search for finer-scale, subtle signal patterns, which multivariate approaches might detect (Tamir and Thornton, 2018). Our findings are only correlational, as in many fMRI studies, and not definitive for the activations encoding/signaling the variable. Our PPI analysis findings are also correlational. Given our focus on the key variables and assumed process, we may have overlooked other processes occurring simultaneously, such as task readiness or a salience network effect (Seeley et al., 2007; Deco et al., 2015; Uddin, 2015; Tamir et al., 2016). Our parsimonious interpretations remain open to broader accounts. Generally, a task variable is related to a broad account if the account is translated in the task context to something sufficiently similar to the variable. However, it is generally unclear whether the account or variable should come first, especially if the variable is essential. For instance, how are the amygdala activations with others’ choice probability related to the salience or emotional response accounts of the amygdala (Adolphs, 2002; Phelps and LeDoux, 2005; Seeley et al., 2007; Cunningham and Brosch, 2012)? This probability would correlate with subjects’ degree of certainty about others’ actions. The activation signaling the probability might underlie uncertainty-related activations in the amygdala, or such activations might actually underlie this study's observations. Computationally, a challenge is to develop a conceptual and quantitative framework consistently linking broad accounts with various concrete decision-making processes in specific contexts and to validate it in fMRI studies.

We found that PCC and rdlPFC activations significantly correlated with primary and secondary value (or reward magnitude) differences, respectively. This finding indicates separate neural valuation signals for one's own decision-making, distinguishing likely and less likely cases of others’ choices. We found that mPFC activation significantly correlated with the participants’ own decisions in both the main and control trials, regardless of the involvement of predicting others’ choices. The mPFC reportedly helps integrate variables of social information to generate one's choices (Amodio and Frith, 2006; van den Bos et al., 2013; Smith et al., 2014; Hutcherson et al., 2015; Hill et al., 2017; Fukuda et al., 2019). These signals are usually demonstrated as correlates to a single decision variable. In actual social environments, however, we often need to consider both likely and unlikely choices of others before making choices, which might depend on others’ choices. Our findings indicate that such signals correlated with the PCC and rdlPFC activations, apart from mPFC activation for final decisions. The separate primary and secondary valuation are computed as one's own value difference given the predicted, primary (more likely) and secondary (less likely) choices of others and are correlated with the PCC and rdlPFC activations, respectively.

The PCC is considered to be involved in different stages of the decision-making process, such as response for values across tasks and reward modalities (Clithero and Rangel, 2014). In social cognition, the PCC was related to theory of mind, empathy, and morals (Fletcher et al., 1995; Kanske et al., 2017; Hopp et al., 2023). Recent studies emphasized the role of the PCC in situating the self in social interactions (Yeshurun et al., 2021). In our findings, the PCC signals correlated with one's own value difference given the prediction of others’ more likely choices. Intuitively, this variable is ubiquitous broadly for social interactions, suggesting it serves as an essential building block for more complex social functions such as those mentioned above. On the other hand, the dlPFC, as part of the frontal-parietal executive control system, executes and adjusts decisions (Miller and Cohen, 2001; Schmidt et al., 2018). In social cognition, the dlPFC is considered to be related to social norms, showing activations and causal modulations in aversive behaviors toward unfairness and guilt (Knoch et al., 2006; Baumgartner et al., 2011; L. J. Chang et al., 2011; Nihonsugi et al., 2015; Gao et al., 2018). The secondary value difference in this study seems to have a parallel with social norms in modulating choices. The secondary valuation complements the primary valuation, while knowledge of social norms also complements a decision, by adjusting one's own decisions according to the norm. This study identified signals of the particular decision variable in the rdlPFC. It would be intriguing to study what other kinds of social decision variables in clear computational forms are related to the rdlPFC and how they may be related to social norms or adjust one's decision-making.

We also analyzed the functional connectivity and temporal order of the activations discussed above. Our findings suggest that the signals in the left amygdala may help balance the signals of the two value differences in the PCC and rdlPFC to generate actual choices in the mPFC. We found significant functional connectivities of the (left) amygdala activations with the PCC and rdlPFC activations. The effects were opposing each other, consistent with our assumed processes. When the predicted others’ choice probability was high (i.e., easy to predict), the associated amygdala signals were higher. The elevated signals would provide stronger support to and suppression of the PCC and rdlPFC, respectively, resulting in greater reliance on the primary valuation for the choices. Conversely, when the choice probability was low (difficult prediction), the amygdala signals were lower, leading to less support and inhibitions to the PCC and rdlPFC, respectively, and together resulting in facilitated use of both primary and secondary valuations. We also found significantly positive connectivities of the PCC and rdlPFC activations with the mPFC activations. Temporally, activations were earlier in the amygdala and later in the mPFC and somewhat in the middle in the PCC and rdlPFC, coherent with the assumed processes. Taken together, these findings suggest that these signals could form a cascade of processing: what choices are likely made by others (left amygdala) are fed to weigh the secondary scenario (rdlPFC), in addition to the primary scenario (PCC), and, then, the two signals contribute to generate a choice (mPFC), although these interactions do not have to be feedforward only and may involve recurrent interactions, for instance.

In conclusion, this study explored behavior and neural computations in a situation where individuals make decisions while predicting both likely and less likely choices of others. Because we consider these to be essential for decision-making when interacting with other individuals, we believe that the neural signals identified here will be widely applicable across a broad domain of social cognition and may underlie more complex social behavior and neural correlates.
